# Impact of Oncological Treatment on Quality of Life in Patients with Head and Neck Malignancies: A Systematic Literature Review (2020–2025)

**DOI:** 10.3390/curroncol32070379

**Published:** 2025-06-30

**Authors:** Raluca Grigore, Paula Luiza Bejenaru, Gloria Simona Berteșteanu, Ruxandra Ioana Nedelcu-Stancalie, Teodora Elena Schipor-Diaconu, Simona Andreea Rujan, Bianca Petra Taher, Șerban Vifor Gabriel Berteșteanu, Bogdan Popescu, Irina Doinița Popescu, Alexandru Nicolaescu, Anca Ionela Cîrstea, Catrinel Beatrice Simion-Antonie

**Affiliations:** 1 Department 12-Otorhynolaryngology, Ophthalmology, Faculty of Medicine, “Carol Davila” University of Medicine and Pharmacy, 020021 Bucharest, Romania; raluca.grigore@umfcd.ro (R.G.); simona-gloria.munteanu@drd.umfcd.ro (G.S.B.); ruxandra-ioana@drd.umfcd.ro (R.I.N.-S.); elena-teodora.diaconu@drd.umfcd.ro (T.E.S.-D.); simona-andreea.rujan@drd.umfcd.ro (S.A.R.); bianca-petra.taher@drd.umfcd.ro (B.P.T.); serban.bertesteanu@umfcd.ro (Ș.V.G.B.); bogdan_popescu@umfcd.ro (B.P.); irina-doinita.popescu@drd.umfcd.ro (I.D.P.); alexandru.nicolaescu@umfcd.ro (A.N.); anca-ionela.cirstea@drd.umfcd.ro (A.I.C.); catrinel.simion-antonie@drd.umfcd.ro (C.B.S.-A.); 2 Otorhinolaryngology Department, “Colțea” Clinical Hospital, 030171 Bucharest, Romania; 3 Otorhinolaryngology Department, “Dr. Carol Davila” Central Military Emergency University Hospital, 010825 Bucharest, Romania

**Keywords:** quality of life, head and neck malignancies, oncological treatment, PROM, rehabilitation, de-escalation

## Abstract

**Background**: Quality of life (QoL) is a critical indicator in assessing the success of oncological treatments for head and neck malignancies, reflecting their impact on physiological functions and psychosocial well-being beyond mere survival. Treatments (surgery, radiotherapy, chemotherapy) pose multiple functional and emotional challenges, and recent advancements underscore the necessity of evaluating post-treatment QoL. **Objective**: This literature review investigates the impact of oncological treatment on the QoL of patients with malignant head and neck cancers (oral, oropharyngeal, hypopharyngeal, laryngeal) and identifies factors influencing their QoL index. **Methodology**: Using a PICO framework, studies from PubMed Central were analyzed, selected based on inclusion (English publications, full text, PROM results) and exclusion criteria. The last research was conducted on 6 April 2025. From 231 identified studies, 49 were included after applying filters (MeSH: “Quality of Life,” “laryngeal cancer,” “oral cavity cancer,” etc.). Data were organized in Excel, and the methodology adhered to PRISMA standards. **Results**: Treatment Impact: Oncological treatments significantly affect QoL, with acute post-treatment declines in functions such as speech, swallowing, and emotional well-being (anxiety, depression). Partial recovery depends on rehabilitative interventions. Influencing Factors: Treatment type, disease stage, socioeconomic, and demographic contexts influence QoL. De-escalated treatments and prompt rehabilitation improve recovery, while complications like trismus, dysphagia, or persistent hearing issues reduce long-term QoL. Assessment Tools: Standardized PROM questionnaires (EORTC QLQ-C30, QLQ-H&N35, MDADI, HADS) highlighted QoL variations. Studies from Europe, North America, and Asia indicate regional differences in outcomes. **Limitations**: Retrospective designs, small sample sizes, and PROM variability limit generalizability. Multicentric studies with extended follow-up are recommended. **Conclusions**: Oncological treatments for head and neck malignancies have a complex impact on QoL, necessitating personalized and multidisciplinary strategies. De-escalated therapies, early rehabilitation, and continuous monitoring are essential for optimizing functional and psychosocial outcomes. Methodological gaps highlight the need for standardized research.

## Simple Summary

Head and neck cancers, affecting areas like the mouth, throat, and voice box, are treated with surgery, radiation, or chemotherapy; however, these treatments can reduce quality of life by impacting speech, swallowing, and emotional well-being. This review shows that while treatments help control cancer, they often cause temporary declines in daily functions, with recovery depending on rehabilitation like swallowing exercises or nutritional support. Factors such as cancer stage, income, and mental health affect recovery. Newer, gentler treatments and early rehabilitation improve outcomes, especially for certain patients. However, studies often have limitations like small sample sizes, making results less certain. Personalized treatment plans, combining medical care with support for nutrition and mental health, are crucial for improving patients’ lives. Future research should use consistent methods to better understand long-term effects and tailor treatments to individual needs.

## 1. Introduction

QoL is a critical indicator in assessing the success of oncological treatments, providing insights into their impact beyond mere survival. In the context of malignant head and neck neoplasms, challenges are multifaceted, with treatment effects—whether surgical, radiotherapy, chemotherapy, or combinations thereof—extending to patients’ physiological and psychosocial domains. Recent advancements in oncology have improved survival rates and heightened expectations for comprehensive functional and emotional recovery, making post-treatment QoL evaluation indispensable.

This literature review aims to synthesize data published over the past five years on the impact of oncological treatments on QoL in patients with malignant head and neck neoplasms. Utilizing a PICO framework—focusing on diagnosed patients, specific interventions (surgery, radiotherapy, chemotherapy), comparison of therapeutic strategies, and outcome evaluation through validated Patient-Reported Outcome Measures (PROMs)—the study seeks to identify factors directly or indirectly influencing QoL indices.

The methodology involved a rigorous selection of studies from PubMed Central, applying clear inclusion criteria (English-language publications, full-text availability, and PROM-based outcomes) and exclusion criteria to ensure a relevant and high-quality synthesis. By analyzing 49 studies, this review aims to provide a comprehensive overview of key findings in the field while highlighting knowledge gaps requiring future research. The results are expected to inform the development of more effective management and rehabilitation strategies tailored to the specific needs of head and neck oncology patients.

Initial findings from the analyzed studies indicate that oncological treatments for head and neck malignancies significantly impact physiological functions and psychosocial well-being. Standardized assessment tools reveal an acute decline in QoL in the immediate post-treatment period, manifested through speech and swallowing difficulties, dysphonia, and increased symptoms of anxiety and depression. Subsequent recovery trends, contingent on applied rehabilitative interventions, suggest partial improvement; however, the absence or delay of rehabilitation strategies may lead to persistent complications that negatively affect patients’ overall well-being.

Another key discussion in the literature pertains to significant outcome variations influenced by factors such as treatment type, disease stage, and the demographic and socioeconomic context of the studied populations. Studies from different regions (Europe, North America, Asia) demonstrate that patients receiving de-escalated treatments or prompt rehabilitation programs recover more rapidly to pre-treatment QoL levels compared to those without such interventions. Persistent adverse effects, such as trismus, dysphagia, or hearing impairments, remain critical factors contributing to long-term QoL deterioration, underscoring the need for continuous monitoring and personalized interventions.

Methodological discussions highlight limitations inherent in various studies, including retrospective designs, small sample sizes, and variability in PROM questionnaire application. These issues raise concerns about generalizability and uniform result interpretation, emphasizing the need for multicenter studies with extended follow-up and standardized methodological criteria. Consequently, the development of integrated evaluation frameworks is essential to enable a holistic synthesis of treatment effects on QoL, ultimately contributing to the optimization of therapeutic strategies and enhanced post-treatment management in head and neck oncology.

*Objective:* This literature review was conducted to investigate the research question: What is the impact of oncological treatment on the QoL of cancer patients diagnosed with malignant head and neck neoplasms, and what factors influence their QoL index? The review focused on studies involving the most common head and neck cancers: oral cavity and lip cancer, oropharyngeal cancer, hypopharyngeal cancer, and laryngeal cancer.

## 2. Materials and Methods

The methods used for data collection, outcome definitions, variable extraction, risk of bias assessment, and result synthesis are described below.

### 2.1. Data Collection Methods

The literature search was conducted using PubMed Central, targeting studies published between 1 January 2020 and 6 April 2025, involving human subjects. The search strategy employed Medical Subject Headings (MeSH) terms and keywords: “Quality of life” [MeSH] OR “Health-related Quality of Life” OR “QoL” AND “laryngeal cancer” OR “lip cancer” OR “oral cavity cancer” OR “oropharyngeal cancer” OR “hypopharyngeal cancer”. No automation tools were used in the search process.

Two reviewers independently screened titles and abstracts to identify eligible studies. Disagreements were resolved through discussion to reach a consensus. Full-text articles were retrieved for studies meeting initial inclusion criteria. Data extraction was performed collaboratively by the two reviewers, with each report independently assessed to ensure accuracy. No direct contact was made with study investigators to obtain or confirm data.

### 2.2. Inclusion and Exclusion Criteria

Studies were included if they met the following criteria: (1) were published in English; (2) had full-text availability; (3) focused on the specified head and neck cancers; and (4) used PROMs to assess Health-related QoL (HrQoL). Exclusion criteria included studies that did not use PROMs, were not published in English, or lacked full-text access. Initially, 231 studies were identified. After screening titles and abstracts, 106 studies were excluded (30 on laryngeal cancer, 15 on oral cavity and lip cancer, 48 on oropharyngeal cancer, and 13 on hypopharyngeal cancer) for not meeting PROM-based criteria. Following full-text review, 76 additional studies were excluded, resulting in 49 studies included for analysis.

### 2.3. Outcomes and Variables

The primary outcome was HrQoL as measured by PROMs, including all validated instruments (e.g., EORTC QLQ-C30, QLQ-H&N35, FACT-H&N) across all reported time points and analyses. Secondary outcomes included factors influencing QoL, such as physical functioning, psychological well-being, social functioning, and symptom burden (e.g., pain, swallowing difficulties). All results compatible with these outcome domains were sought from each study.

Additional variables extracted included:

Participant characteristics: Age, sex, cancer stage, and comorbidities.

Intervention characteristics: Type of oncological treatment (surgery, radiotherapy, chemotherapy, or combined modalities).

Study characteristics: Study design, sample size, and funding sources.

Missing or unclear information (e.g., unspecified PROM versions or incomplete participant demographics) was noted, and assumptions were made that missing data did not significantly alter the study’s findings unless stated otherwise.

### 2.4. Risk of Bias Assessment

The risk of bias in included studies was assessed using the Newcastle–Ottawa Scale (NOS) for observational studies and the Cochrane Risk of Bias Tool for randomized controlled trials, where applicable. Two reviewers independently evaluated each study, with disagreements resolved through discussion. No automation tools were used in this process.

### 2.5. Data Synthesis and Presentation

Data were synthesized narratively, focusing on thematic analysis of HrQoL outcomes and influencing factors. No meta-analysis was performed due to the heterogeneity of PROM instruments, study designs, and reported outcomes. Results were tabulated to summarize study characteristics, PROMs used, QoL outcomes, and influencing factors. Visual displays, such as tables, were used to present individual study findings and synthesized results.

### 2.6. Effect Measures

For studies reporting quantitative HrQoL outcomes, effect measures included mean differences in PROM scores (e.g., baseline vs. post-treatment) or standardized mean differences when different PROMs were used. Qualitative findings were summarized descriptively to identify common themes (e.g., impact of treatment type on QoL).

### 2.7. Eligibility for Synthesis

Studies were grouped for synthesis based on cancer type (oral cavity/lip, oropharyngeal, hypopharyngeal, laryngeal) and treatment modality. Eligibility for synthesis was determined by tabulating study characteristics and ensuring alignment with the review’s focus on PROM-based HrQoL outcomes.

### 2.8. Handling Missing Data

Missing summary statistics (e.g., standard deviations for PROM scores) were noted, and where possible, data were imputed using standard methods (e.g., assuming symmetry in reported ranges). No data conversions were required.

### 2.9. Heterogeneity and Sensitivity Analyses

Heterogeneity in study results was explored narratively by comparing study populations, treatment modalities, and PROM instruments. No formal subgroup analysis or meta-regression was conducted due to the narrative approach. Sensitivity analyses were not performed, as the synthesis was qualitative.

### 2.10. Risk of Bias Due to Missing Results

To assess potential reporting biases, we examined whether studies reported all expected PROM outcomes and noted any selective reporting. Funnel plots or formal statistical tests for publication bias were not used due to the narrative synthesis.

### 2.11. Certainty of Evidence

The certainty of evidence for HrQoL outcomes was assessed using the GRADE (Grading of Recommendations Assessment, Development and Evaluation) approach, considering risk of bias, inconsistency, imprecision, and indirectness. The overall certainty was summarized narratively for each outcome.

**Figure 1:**
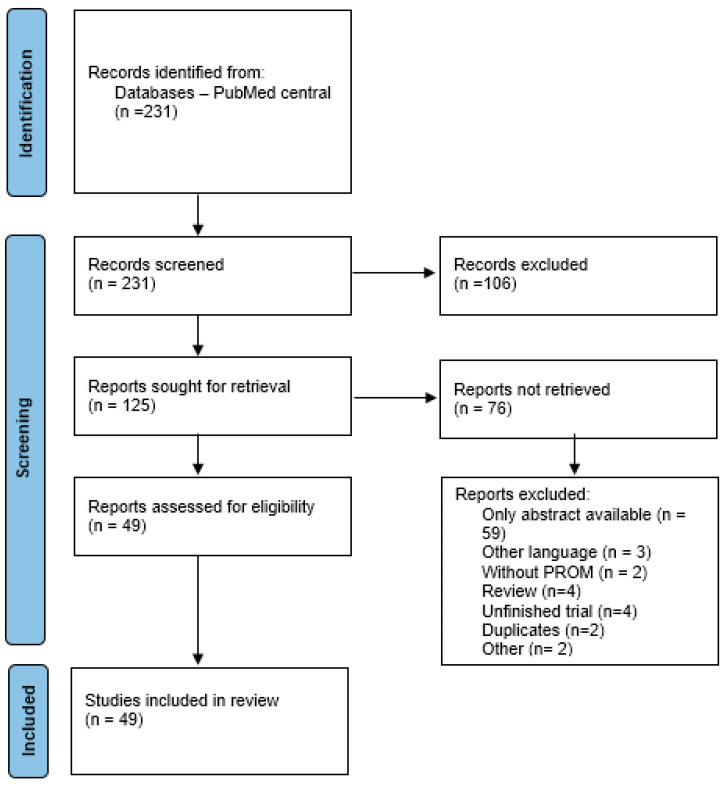
Identification of studies via databases and registers: PRISMA 2020 flow diagram [1].

The methodology is illustrated in the accompanying flowchart and adapted PRISMA diagram (Figure 1). Data were organized using Microsoft Excel. Mendeley software version 1.19.4 was used for identifying duplicates and organizing References.

## 3. Results

Table 1 lists the main types of studies included in this review: 14 cross-sectional observational studies, 1 case series, 1 completed clinical trial with available PROM, 1 national study, 18 prospective studies, 8 randomized controlled trials, 4 retrospective studies, 1 sub-study of a randomized trial with available PROMs, and 1 survey study.

Regarding sample size, the number of patients included in the studies ranged from a minimum of 3 to a maximum of 2171, as shown in Table 1. The mean patient age was 60.7 years, with a male predominance of 79.65%. Demographic distribution was not available in all studies.

Treatment types, as summarized in Table 2, included various approaches, with or without rehabilitation measures.

QoL assessment for patients with head and neck neoplasms was conducted using standardized instruments, with the main accredited questionnaires listed in Table 3.

### 3.1. Main Results of the Studies

***QoL Outcomes:*** The impact of head and neck cancer (HNC) treatments on HrQoL is a recurring theme across multiple studies. A 2021 UK study with three patients reported that salvage robotic-assisted resection with free flap reconstruction achieved complete lesion clearance and good QoL outcomes, despite one patient developing a tracheocutaneous fistula (Williamson A et al., 2021) [2]. Similarly, a 2022 US study involving 79 patients with HPV-positive oropharyngeal cancer found that de-escalated adjuvant chemoradiation resulted in low long-term toxic effects, with QoL returning to baseline levels and no long-term feeding tube dependence (Price K et al., 2022) [3]. However, challenges persist, as a 2023 US study of 880 patients noted that 64.4% experienced hearing loss and tinnitus, significantly associated with worse HrQoL (Aggarwal P et al., 2023) [4]. A 2022 Denmark and Sweden study with 172 patients reported that 46% had moderate/severe dysphagia, 57% had voice problems, and psychological distress (HADS score ≥ 15) and frailty (G8 score < 15) were linked to poorer QoL (Wulff NB et al., 2022) [5]. In Taiwan, a 2024 study of 461 patients found that higher EORTC QLQ-HN35 scores were associated with increased risks of incomplete chemoradiation, emergency visits, hospitalizations, and toxicities, while lower scores correlated with better overall survival (OS) and disease-free survival (DFS) (Hung CY, 2024) [6]. HPV-positive patients exhibited better pre-treatment QoL but greater deterioration during treatment, with faster recovery compared to HPV-negative patients (Korsten LHA et al., 2021) [7]. Treatment type also influenced outcomes, with surgery plus RT causing worse QoL scores than surgery alone, though many returned to baseline after three months (Goiato MC et al., 2020) [8].

***Swallowing and Voice Function:*** Swallowing and voice impairments significantly affect HNC patients’ QoL. A 2021 Ethiopian study of 102 patients reported a mean MDADI score of 53.29, indicating impaired swallowing-related QoL, particularly in female patients, those with low income, advanced tumor stage, or laryngeal cancer (Yifru TA et al., 2021) [9]. In Spain, a 2022 study with 21 patients found that 100% of patients had swallowing efficacy impairments, 85.5% had safety impairments, and 78% showed voice changes with altered CAPE-V attributes, leading to reduced QoL (Alvarez-Marcos C et al., 2022) [10]. A 2023 Danish study of 44 patients noted significant swallowing function improvements from 1 to 3 years post-treatment, with TORS patients showing better safety scores and QoL compared to RT patients, who experienced persistent QoL decline (Scott SI et al., 2023) [11]. In China, a 2024 study of 21 patients reported good swallowing (mean MDADI score 92.67) and voice (mean VHI-10 score 7.14) recovery post-laryngeal cancer treatment (Liu T et al., 2024) [12]. Tracheoesophageal voice prostheses improved socio-emotional and functional outcomes compared to esophageal speech, though fistula complications negatively impacted QoL (Cocuzza S et al., 2020) [13]. The EP-SHI and HoCoS tools were validated for assessing speech-related QoL and communication impairments, showing strong reliability and validity (Guimaraes I et al., 2021; Balaguer M et al., 2023) [14,15].

***Psychological Distress and Nutritional Impact:*** Psychological distress and nutritional challenges are critical determinants of QoL in HNC patients. A 2022 Canadian study of 146 patients found that HPV-negative patients had higher anxiety and depression at diagnosis, while HPV-positive patients showed increased vulnerability post-treatment, with major depressive disorder significantly impacting QoL (Henry M et al., 2022) [16]. A 2023 US study of 115 patients reported that anxiety and depression were inversely correlated with all QoL domains, with younger age, higher income, and early-stage cancer linked to better physical functioning (Andersen LP et al., 2023) [17]. In Finland, a 2024 study of 203 patients noted higher depression rates and lower socioeconomic status in HNC patients compared to the general population, though psychosocial factors did not influence patient delay (Atula M, 2024) [18]. Nutritional supplements reduced malnutrition (40.2% prevalence) and supported QoL recovery in a 2021 Indian study of 97 patients (Pingili S et al., 2021) [19]. A 2023 Thai study of 72 patients found that Nutri-PEITC Jelly intake improved HrQoL and progression-free survival (PFS) without serious adverse events (Lam-Ubol A et al., 2023) [20].

***Oncological and Functional Outcomes:*** Oncological and functional outcomes vary by treatment and patient characteristics. A 2022 French study of 53 patients reported preoperative, 1-year, and 2-year MDADI scores of 71.4, 64.3, and 57.5, respectively, with 97.1% decannulation and 59% two-year OS (D’Andréa G et al., 2022) [21]. In China, a 2023 study of 64 patients reported three-year OS of 60.7%, five-year OS of 47.3%, and 78.1% satisfactory swallowing function, while a 2022 study of 122 patients noted five-year OS and DFS of 40.0% and 36.1%, respectively, with local–regional recurrence and distant metastasis impacting survival (Li WX et al., 2022, 2023) [22]. A 2023 UK and Ireland study of 112 patients found that dysphagia-optimized IMRT (DO-IMRT) improved MDADI scores (77.7 vs. 70.6) compared to standard IMRT, with lower radiation doses to pharyngeal constrictors (Nutting C et al., 2023) [23]. Trismus, reported by 31% of 892 US patients, was associated with increased dysphagia and feeding tube dependence, though jaw stretching exercises reduced prevalence (Cardoso RC et al., 2021) [24]. Sentinel lymph node biopsy (SLNB) offered better short-term shoulder function compared to elective neck dissection (END) (van Hinte G et al., 2021) [25]. A 2021 US study of 80 patients highlighted that extensive tongue resection was strongly linked to poor QoL outcomes (Jimenez JE et al., 2021) [26].

The main results are compiled in Table 4.

### 3.2. Main Conclusions of the Studies

***QoL Outcomes****:* Numerous studies underscore the profound impact of HNC treatments on HrQoL. A 2021 study from the UK reported that oropharyngeal squamous cell carcinoma (OPSCC) patients undergoing ORS-assisted resection with radial forearm free flap (RFFF) reconstruction achieved good oncological and QoL outcomes, despite postoperative complications (Williamson A et al., 2021) [2]. Similarly, a 2022 US study on de-escalated adjuvant therapy for oropharyngeal cancer demonstrated excellent swallow outcomes and preserved QoL with reduced long-term toxic effects (Price K et al., 2022) [3]. However, persistent impairments were noted, with a 2022 study from Denmark and Sweden reporting that voice problems, dysphagia, depression, and anxiety were independently associated with lower HrQoL in hypopharyngeal and laryngeal cancer patients (Wulff NB et al., 2022) [5]. In a 2024 Taiwanese study, pre-treatment HrQoL, assessed via QLQ-HN35, was a significant predictor of treatment-related complications, tolerance, and survival, with higher scores linked to increased complications (Hung CY, 2024) [6]. HPV-positive oropharyngeal cancer patients generally exhibited better QoL recovery compared to HPV-negative patients, emphasizing the need for tailored supportive care based on HPV status (Korsten LHA et al., 2021) [7]. Additionally, a 2020 UK study highlighted baseline HrQoL as a prognostic indicator for survival, advocating its integration into clinical care (Rogers SN et al., 2020) [38]. Treatment modalities also influenced QoL, with surgery plus RT causing greater morbidity than surgery alone, though recovery trends were observed within three months (Goiato MC et al., 2020) [8].

***Swallowing and Voice Function***: Swallowing and voice impairments significantly affect HNC patients’ QoL. A 2021 study from Ethiopia reported that dysphagia substantially impacted swallowing-related QoL, recommending routine swallowing assessments (Yifru TA et al., 2021) [9]. In Brazil, a 2024 study found that SCPL preserved laryngeal function while ensuring oncological safety, and a 2025 study confirmed that fiberoptic endoscopic evaluation of swallowing (FEES) effectively guided early rehabilitation post-laryngectomy (Liu T et al., 2024; Jia L et al., 2025) [12,41]. Tracheoesophageal prostheses were identified as the gold standard for vocal rehabilitation, improving QoL in laryngectomy patients, though fistula-related complications required careful management (Souza FGR et al., 2020; Cocuzza S et al., 2020) [13,30]. In contrast, electrolarynx use was a viable alternative, positively impacting QoL (Monte LEFD et al., 2024) [29]. A 2023 Danish study noted better long-term swallowing function and QoL in TORS patients compared to RT patients, who showed persistent QoL decline despite functional recovery (Scott SI et al., 2023) [11]. Asymptomatic swallowing disorders were common post-chemoRT, with FEES and V–VST proving useful for detection (Alvarez-Marcos C et al., 2022) [10]. The EP-SHI and HoCoS tools were validated as reliable measures for assessing speech-related QoL and communication impairments, respectively (Guimaraes I et al., 2021; Balaguer M et al., 2023) [14,15].

***Psychological Distress and Nutritional Support****:* Psychological distress and nutritional challenges are critical factors influencing QoL in HNC patients. A 2022 Canadian study found that HPV-negative patients experienced greater psychological distress at diagnosis, while HPV-positive patients required equal support post-treatment, with major depressive disorder, anxiety, and depression significantly affecting QoL (Henry M et al., 2022) [16]. A 2024 Finnish study noted that while psychosocial factors did not influence patient delay, lower socioeconomic status and higher depression rates were prevalent among HNC patients (Atula M, 2024) [18]. Nutritional interventions were vital, with a 2021 Indian study highlighting that nutritional supplements reduced malnutrition, aiding symptom recovery and QoL improvement (Pingili S et al., 2021) [19]. Similarly, a 2023 Thai study found that Nutri-PEITC Jelly intake improved QoL and progression-free survival (PFS) in advanced oral and oropharyngeal cancer patients (Lam-Ubol A et al., 2023) [20]. A 2022 French study emphasized the role of dental rehabilitation, psychological support, and nutritional measures in elderly oropharyngeal cancer patients, noting a negative correlation between patient concerns and QoL (Bozec A et al., 2022) [27].

***Oncological and Functional Outcomes****:* Oncological control and functional outcomes varied by treatment and disease characteristics. A 2022 French study on robotic-assisted salvage surgery for oropharyngeal cancer reported satisfactory QoL, good functional sequelae, and favorable oncological outcomes compared to historical approaches (D’Andréa G et al., 2022) [21]. In China, surgery-oriented comprehensive treatment for hypopharyngeal and laryngeal cancer achieved good swallowing function without compromising oncological control, though surgical defect size, local–regional recurrence, and distant metastasis were independent factors impacting survival and swallowing function (Li WX et al., 2022) [48]. A 2023 UK and Ireland study found that dysphagia-optimized intensity-modulated RT (DO-IMRT) improved patient-reported swallowing function compared to standard IMRT, suggesting it as a new standard of care (Nutting C et al., 2023) [23]. Trismus, prevalent in advanced oropharyngeal cancer, was associated with tumor subsite (tonsil) and concurrent CT, negatively impacting QoL (Cardoso RC et al., 2021) [24]. SLNB offered better short-term shoulder function compared to elective neck dissection (END) (van Hinte G et al., 2021) [25]. Additionally, a 2021 US study highlighted that the extent of tongue resection was strongly associated with poor QoL outcomes in oral cavity cancer patients, emphasizing the need for multidisciplinary postoperative care (Jimenez JE et al., 2021) [26].

Table 5 outlines the main conclusions of each study in relation to the affected region and reported limitations.

### 3.3. QoL and Functional Outcomes Across Studies: Risk of Bias Assessment

***Timeframes:*** Outcomes ranged from immediate post-treatment (e.g., hospital stay, 1 week) to long-term follow-up (up to 16 years). Short-term studies (≤12 months) often reported QoL declines, with partial recovery by 3–12 months [8,11]. Long-term studies (≥1 year) showed persistent dysphagia, xerostomia, and voice issues, though some patients achieved good QoL [4,27].

***Outcomes:***

✓ Swallowing: Subclinical swallowing disorders were common, detected by tools like MDADI, V-VST, and FEES, significantly impacting QoL, especially in laryngeal/hypopharyngeal cancers [10,40].
✓ Voice: Subclinical voice disorders were frequent post-treatment; rehabilitation improved outcomes in some cases [7,37].
✓ QoL: HrQoL was influenced by treatment modality, tumor stage, and psychosocial factors. HPV-related oropharyngeal cancers often had better QoL than non-HPV cases [7,16]. Malnutrition, trismus, and xerostomia reduced QoL [19,24].
✓ Psychosocial Factors: Anxiety, depression, and low health literacy correlated with worse QoL, but psychosocial factors did not consistently predict treatment delays [17,18].

***Treatment Modalities:***

✓ Surgery: Free-flap reconstruction and robotic-assisted surgeries (e.g., TORS) were feasible but showed mixed QoL outcomes due to small samples and bias [2,21]. Laryngectomy patients’ QoL varied with vocal rehabilitation methods [13,30].
✓ RT/ChemoRT: Dose de-escalation in HPV-positive oropharyngeal cancer reduced toxicity but not swallowing/QoL impairments [3]. Dysphagia-optimized IMRT improved swallowing compared to standard IMRT [23].
✓ Adjuvant Therapies: Swallowing exercises, voice rehabilitation, and acupuncture moderately improved function and QoL, though evidence was limited [43,44,45].
✓ Novel Interventions: Nutri-PEITC jelly enhanced progression-free survival and QoL in advanced cases [20].

***GRADE Certainty of Evidence:***

✓ Most studies were rated very low (31/49) or low (12/49) due to:
  - Risk of Bias: Non-randomized designs, lack of blinding, selection/survival bias, self-reported outcomes, and small samples [5,28].
  - Inconsistency: Mixed findings and few comparable trials [34].
  - Indirectness: Limited generalizability from single-center studies [15].
  - Imprecision: Small samples, wide confidence intervals, low event rates [35].
✓ Moderate certainty (6/49) occurred in studies with larger samples or validated tools (e.g., MDADI, EORTC-QLQ) [38,42].
✓ High certainty was rare, seen in one RCT sub-analysis with low bias and precise estimates [46].

***Key Predictors of QoL:***

- Demographic/Clinical: Advanced tumor stage, female gender, low income, and extensive surgery predicted worse QoL [25,26].
- Treatment-Related: Multimodal treatments (surgery + RT) caused worse short-term QoL than single-modality treatments [25].
- Functional: Persistent dysphagia, voice impairment, trismus, and malnutrition strongly reduced QoL [31,32].
- Biological: Oxidative stress markers (e.g., SOD, MDA) predicted complications and QoL post-surgery [39].

***Limitations and Implications***

- Data Limitations: Small samples, cross-sectional designs, and self-reported outcomes limit causal inferences. Long-term data are sparse [14,29].
- Clinical Implications: Multidisciplinary care (swallowing/voice rehab, nutritional support, psychosocial interventions) is critical for QoL optimization. HPV status and de-escalation strategies may improve oropharyngeal cancer outcomes [7,49].
- Research Needs: Larger, randomized trials with standardized measures (e.g., MDADI, EORTC-QLQ) and extended follow-ups are needed [33].

***Risk of bias*** in the included randomized controlled trials was assessed using the Revised Cochrane Risk of Bias Tool (RoB 2), which evaluates five key domains: bias arising from the randomization process, deviations from intended interventions, missing outcome data, measurement of the outcome, and selection of the reported result [51]. Each domain is judged as “low risk,” “some concerns,” or “high risk” of bias [51]. The overall risk of bias for each study was determined according to Cochrane guidelines [51].

Overall Trends:

➢ Seven of eight RCTs (Nutting, Lam-Ubol, Hajdú, Karlsson, Jansen, Johansson, Theurer) have some concerns overall, driven by:
➢ Domain 2 (Deviations): Lack of patient blinding in rehabilitation (Hajdú, Karlsson, Jansen, Johansson), surgical/radiation (Nutting, Theurer), or nutritional trials (Lam-Ubol is an exception due to placebo).
➢ Domain 3 (Missing Data): Potential dropout in cancer/rehabilitation trials, except Nutting (phase 3 rigor).
➢ Domain 4 (Outcome Measurement): Subjective outcomes (QoL, swallowing, voice) with unblinded patients, except Lam-Ubol (blinded QoL/PFS).
➢ Xuewei et al. (#44) have a high risk overall, due to high risk in Domains 2 and 4 (no blinding, subjective QoL) and some concerns in Domains 1, 3, and 5, reflecting weaker methodology in acupuncture trials.

Strengths:

➢ Domain 1 (Randomization): Seven RCTs are low risk, indicating robust randomization in high-quality journals (Lancet Oncol, Oral Oncol, Head Neck). Xuewei is an exception (Some concerns).
➢ Domain 5 (Reported Result): Six RCTs are low risk, suggesting protocol adherence, especially in multicenter/phase 3 trials. Xuewei and Johansson have some concerns due to potential unregistered protocols.

Weaknesses:

➢ Domain 2 (Deviations): Most RCTs (6/8) have some concerns due to impractical blinding in non-pharmacological interventions. Xuewei is high risk (no blinding, analytical issues).
➢ Domain 4 (Outcome Measurement): Six RCTs have some concerns due to subjective outcomes and unblinded patients. Xuewei is high risk (unvalidated QoL). Lam-Ubol is low risk (blinded, objective PFS).
➢ Domain 3 (Missing Data): Seven RCTs have some concerns due to dropout risks in cancer trials. Nutting is low risk (phase 3 rigor).
➢ Non-RCTs: Five studies require NOS or ROBINS-I, not RoB 2, limiting direct comparison with RCTs.

### 3.4. Implications for Systematic Review

Evidence Quality: RCTs with some concerns (7/8) are usable in meta-analyses, but biases (blinding, subjective outcomes) warrant cautious interpretation. Xuewei’s high risk suggests exclusion or sensitivity analysis.

## 4. Discussion

The analyzed results demonstrate significant advancements in the therapeutic management of head and neck cancer, highlighting both oncologic progress and improvements in post-treatment HrQoL. The anatomical regions most frequently affected by malignant neoplasms in the head and neck include the oral cavity, oropharynx, hypopharynx, and larynx. Consequently, quantifying an overall HrQoL index for patients with HNC remains challenging.

**Oral Cavity**

**Surgery**

Surgical approaches, including free tissue reconstruction and split-thickness skin graft (STSG), significantly impact HrQoL. Extensive tongue resection strongly predicts poor QoL, mediating other defect characteristics [26]. STSG suits early-stage floor of mouth carcinoma, but anterior floor cases may require alternative reconstructions [28]. SLNB offers superior short-term shoulder function and cost-effectiveness compared to elective neck dissection [25]. Discrepancies between patient-reported and clinician-rated outcomes underscore the need for PROM in routine care [36].

**Combination Therapy and RT**

Combining surgery with RT exacerbates short-term QoL declines, particularly in oral function, though recovery occurs within months [8]. RT negatively impacts implant survival (*p* < 0.00001), with dentition status and implant timing linked to recurrence and adverse effects [52]. Concurrent chemoradiation results in an improvement of over 20% in overall survival compared to radiation therapy alone [53].

**Rehabilitation**

Oral rehabilitation is critical for optimizing HrQoL, with systematic reviews emphasizing its integration into treatment protocols to enhance functional outcomes [52]. Nutritional supplements reduce malnutrition (40.2% prevalence), supporting recovery [19]. Swallowing assessments (e.g., MDADI) are essential for addressing dysphagia-related QoL declines [9].

**Oropharynx**

**Surgery**

TORS with RFFF reconstruction yields favorable oncologic and HrQoL outcomes, with acceptable complications [2,54]. TORS patients show better long-term swallowing and QoL compared to RT-treated patients, despite short-term declines (3–6 months), recovering by 12 months [32,55]. Advanced T-stage (>9.35 cm^3^) predicts worse swallowing and higher PEG placement rates [56]. Severe dysphagia remains a well-recognized complication following OPSCC surgery, even after TORS [57].

**RT**

Dysphagia-optimized intensity-modulated RT (DO-IMRT) improves swallowing function over standard IMRT, emerging as a potential standard of care [23]. Proton therapy (IMPT) avoids additional swallowing toxicity [40]. De-escalated RT in HPV-positive OPSCC reduces long-term toxicities, preserving QoL [3]. Audiological monitoring is crucial, as hearing loss impairs HrQoL [4].

**CT and Combination Therapy**

Concurrent chemoradiation increases swallowing morbidity, particularly when combined with surgery, though HPV-positive patients recover faster [7,8]. A logarithmic dose-toxicity relationship (3.4% increased dysphagia risk per gray) highlights the need for dose optimization [54,58].

**Rehabilitation and Novel Interventions**

Nutri-PEITC Jelly enhances QoL and progression-free survival in advanced cases [20]. Guided self-help exercises improve swallowing and communication, with early intervention being most effective [42]. Validated PROMs (EP-SHI, HoCoS, HN-LEF SI) ensure comprehensive assessment of speech, communication, and lymphedema [14,15,33]. Nutritional support is vital for both HPV-positive and HPV-negative patients [34].

**Psychological Distress**

Advanced stage, low income, and anxiety/depression predict poorer QoL, requiring pre-treatment screening [16]. HPV-negative patients face greater distress at diagnosis, while HPV-positive patients need post-treatment support for depression and relationship issues [16]. Psychological and nutritional care is critical for elderly patients [27].

**Larynx and Hypopharynx**

**Surgery**

SCPL and laryngeal preservation surgery balance oncologic control and function in early-stage disease [13,22]. TL yields worse HrQoL than SCPL, with TEP improving outcomes despite fistula complications [13,30,45,47,59]. Electrolarynx is still a viable alternative [29]. Surgical defect size and recurrence negatively impact QoL and swallowing [12].

**RT**

RT causes subclinical voice disorders and dysphagia, reducing QoL for up to 24 months [10,37]. Endoscopic laser-assisted surgery and RT for early stages show comparable outcomes, necessitating standardized assessments [60].

**CT and Combination Therapy**

Multimodal treatments increase symptom burden, with oxidative stress from extensive surgery worsening QoL [12,39]. CRT exacerbates dysphagia, requiring long-term rehabilitation [12].

**Rehabilitation**

Voice rehabilitation post-RT improves communication and is cost-effective [45,47]. Acupuncture with swallowing exercises enhances QoL post-surgery [44]. Guided self-help programs and FEES-guided rehabilitation improve swallowing in TL patients [41,43,46]. Swallowing exercises, supported by RCTs, benefit multimodal treatment patients [61]. SLNB reduces complications in metastatic lymphadenopathy but lacks clear HrQoL benefits [62].

**Psychological Distress**

Dysphagia, voice issues, depression, and anxiety significantly reduce HrQoL, with mild symptom burden in TL patients [5,63]. Socioeconomic status and depression warrant clinical attention [18].

**Cross-Site Findings**

Pre-treatment HrQoL (EORTC QLQ-HN35) predicts treatment tolerance and survival, guiding clinical decisions [6,38]. Advanced stage, female gender, low income, and multimodal treatments predict worse QoL [9,16]. Trismus (31% prevalence) and malnutrition (40.2%) impair function across sites [19,24]. Multidisciplinary care, integrating swallowing/voice rehabilitation, nutritional support, and psychological interventions, is essential [9,27,31]. Validated tools ensure accurate monitoring [33,49].

**Novelty**

The novelty of our review lies in emphasizing the importance of using validated assessment tools, the need for improved study designs, and providing a comprehensive overview of the outcomes of various oncological treatments for head and neck cancer. It also highlights strategies for optimizing post-treatment outcomes, refining surgical techniques, and implementing RT and CT protocols. Most literature reviews focus on one single anatomical subsite or one topic. It increasingly emphasizes the importance of rehabilitation strategies and their timely implementation. De-escalated therapeutic approaches, particularly in adjuvant therapy, have proven effective, with reduced radiation doses leading to improved swallowing function and minimized long-term toxicities, contributing to satisfactory HrQoL. Continuous monitoring, including audiological assessments, is emphasized to detect hearing impairments early, as unmanaged impairments may adversely affect long-term outcomes. Late adverse effects, including vocal disorders (often subclinical post-CT or RT), swallowing disorders (including asymptomatic cases detected by fiberoptic endoscopic evaluation of swallowing and volume–viscosity swallow test [V-VST]), trismus, depression, and anxiety, significantly impact HrQoL. Evaluation using validated tools is essential for accurately monitoring treatment effects on vocal and swallowing functions, as well as fibrosis and lymphedema.

From the perspective of post-resection reconstruction, the extent of tongue resection and the choice of reconstruction technique significantly influence functional outcomes and HrQoL. Minimally invasive surgical approaches, such as robotic-assisted surgery, offer advantages in improving functional recovery and reducing complications compared to traditional methods. Integrated supportive interventions, including nutritional supplementation, dental rehabilitation, and psychological counseling, are critical in mitigating the negative impact of treatments, particularly in patients with risk factors such as advanced age, unfavorable socioeconomic status, advanced disease stage, or symptoms of depression and anxiety. Guided self-help programs, vocal rehabilitation therapies, specialized exercises, and complementary interventions such as acupuncture contribute to improved speech and swallowing functions, facilitating faster recovery and enhancing long-term outcomes.

**Limitations**

Although our literature review provides valuable insights into the oncological and QoL outcomes of head and neck cancer treatments, we must acknowledge the selected studies’ limitations—small sample sizes, single-center designs, limited generalizability, selection bias, subjective measures, and short follow-up periods—which restrict the strength and applicability of the findings.

### 4.1. Small Sample Size

Studies Affected: Williamson A et al. (2021) [2], Aggarwal P et al. (2023, 2021) [4,42], Alvarez-Marcos C et al. (2022) [10], Pingili S et al. (2021) [19], Cardoso RC et al. (2021) [24], Bozec A et al. (2022) [27], Guimaraes I et al. (2021) [14], Balaguer M et al. (2023) [15], Larson AR et al. (2021) [28], Monte LEFD et al. (2024) [29], Scott SI et al. (2021, 2023) [11,32], D’Andréa G et al. (2022) [21], Harrowfield J et al. (2021) [34], Ramalingam K et al. (2024) [35], van Hinte G et al. (2021) [25], Balaji H et al. (2024) [36], Tuomi L et al. (2021) [37], Zivkovic A et al. (2024) [39], Liu T et al. (2024) [12], Cocuzza S et al. (2020) [13], Theurer JA et al. (2025) [49], Nakai MY et al. (2021) [50], Zhu X et al. (2022) [44], Johansson M et al. (2020) [47], Jia L et al. (2025) [41].

Implications: Small sample sizes reduce statistical power, leading to wider confidence intervals (e.g., Aggarwal P et al., 2023 [4]) and limiting the ability to detect significant differences or generalize findings. This is particularly problematic in studies assessing rare outcomes or subgroups (e.g., Cardoso RC et al., 2021 [24], for IMPT and PORT patients).

### 4.2. Single-Center Study Design

Studies Affected: Price K et al. (2022) [3], Wulff NB et al. (2022) [5], Alvarez-Marcos C et al. (2022) [10], Pingili S et al. (2021) [19], Cardoso RC et al. (2021) [24], Hung CY et al. (2024) [6], Henry M et al. (2022) [16], Scott SI et al. (2021) [32], D’Andréa G et al. (2022) [21], Deng J et al. (2022) [33], Zivkovic A et al. (2024) [39], Grant SR et al. (2020) [40], Nakai MY et al. (2021) [50], Zhu X et al. (2022) [44], Li WX et al. (2022, 2023) [22,48], Liu T et al. (2024) [12], Nutting C et al. (2023) [23].

Implications: Single-center studies limit generalizability due to institution-specific protocols, patient demographics, and treatment practices. For example, Hung CY et al. (2024) [6] noted limited generalizability due to a single-center design, which may not reflect outcomes in diverse healthcare settings.

### 4.3. Limited Generalizability Due to Regional or Population-Specific Factors

Studies Affected: Bozec A et al. (2022 [27], France-specific protocols), Goiato MC et al. (2020 [8], Brazil-specific protocols), Korsten LHA et al. (2021 [7], Dutch-specific protocols), Scott SI et al. (2023 [11], Denmark-specific protocols), Lam-Ubol A et al. (2023 [20], Thailand-specific protocols), Aggarwal P et al. (2021 [42], Dutch-specific protocols), Yifru TA et al. (2021 [9], Ethiopian context), Rogers SN et al. (2020 [38], UK-based data), Pingili S et al. (2021 [19], Indian population).

Implications: Region-specific treatment protocols, cultural factors, or socioeconomic conditions (e.g., Yifru TA et al., 2021 [9]) limit the applicability of findings to other populations. For instance, modified questionnaires in Pingili S et al. (2021) [19] may not align with standardized global measures.

### 4.4. Potential Selection Bias

Studies Affected: Price K et al. (2022) [3], Cardoso RC et al. (2021) [24], Bozec A et al. (2022) [27], Korsten LHA et al. (2021) [7], Lam-Ubol A et al. (2023) [20], Jimenez JE et al. (2021) [26], Deng J et al. (2022) [33].

Implications: Strict inclusion/exclusion criteria (e.g., Price K et al., 2022 [3]) or sampling from specific clinics (e.g., Jimenez JE et al., 2021 [26]) may exclude patients with different characteristics, skewing results. For example, Jimenez JE et al. (2021) [26] noted potential bias from patients attending survivorship clinics.

### 4.5. Reliance on Self-Reported or Subjective Measures

Studies Affected: Cardoso RC et al. (2021 [24], self-reported trismus), Jimenez JE et al. (2021 [26], subjective PROMs), Andreassen R et al. (2022 [31], self-reported data), Yifru TA et al. (2021 [9], self-reported data), Atula M (2024 [18], recall bias), Ramalingam K et al. (2024 [35], patient-reported outcomes), Balaji H et al. (2024 [36], patient-reported outcomes), Johansson M et al. (2020 [47], recall bias).

Implications: Self-reported measures are prone to recall bias, subjectivity, or discrepancies with clinician assessments (e.g., Balaji H et al., 2024 [36]). This affects the reliability of QoL and functional outcome data.

### 4.6. Cross-Sectional or Retrospective Design

Studies Affected: Jimenez JE et al. (2021 [26], retrospective cross-sectional), Monte LEFD et al. (2024 [29], cross-sectional), Souza FGR et al. (2020 [30], cross-sectional), Andreassen R et al. (2022 [31], cross-sectional), Yifru TA et al. (2021 [9], cross-sectional), Li WX et al. (2022 [48], retrospective), Liu T et al. (2024 [12], retrospective).

Implications: Cross-sectional designs limit causal inferences, while retrospective designs may introduce recall bias or miss preoperative data (e.g., Souza FGR et al., 2020 [30]). These designs cannot capture longitudinal changes in QoL or functional outcomes.

### 4.7. Limited Follow-Up Duration

Studies Affected: Williamson A et al. (2021) [2], Goiato MC et al. (2020 [8], 3 months), Theurer JA et al. (2025) [49], Jia L et al. (2025) [41], Harrowfield J et al. (2021) [34], Zhu X et al. (2022) [44].

Implications: Short follow-up periods fail to capture long-term outcomes, such as late toxicities or recovery trends (e.g., Goiato MC et al., 2020 [8]). This is critical for QoL studies, where long-term impacts are significant.

### 4.8. Missing Data or High Dropout Rates

Studies Affected: Henry M et al. (2022 [16], missing data requiring imputation), Nutting C et al. (2023 [23], missing data requiring imputation), Andersen LP et al. (2023 [17], missing data requiring imputation), Korsten LHA et al. (2021 [7], missing surveys due to death/loss to follow-up), Tuomi L et al. (2021 [37], high dropout rate), Hajdú SF et al. (2022 [43], 25% dropout rate), Karlsson T et al. (2022 [45], reduced participants over time).

Implications: Missing data or high dropout rates reduce the reliability of results and may introduce bias, particularly if dropouts are related to poor outcomes (e.g., Korsten LHA et al., 2021 [7]).

### 4.9. Lack of Preoperative or Baseline Data

Studies Affected: Larson AR et al. (2021 [28], lack of preoperative functional data), Atula M (2024 [18], inability to assess pre-diagnosis psychological status), Nakai MY et al. (2021 [50], lack of preoperative QoL assessments), Zivkovic A et al. (2024 [39], lack of preoperative psychological assessments).

Implications: Without baseline data, it is difficult to attribute changes in QoL or function to treatment rather than pre-existing conditions.

### 4.10. Heterogeneity in Treatment or Patient Characteristics

Studies Affected: Aggarwal P et al. (2023 [4], variability in treatment regimens), Wulff NB et al. (2022 [5], variability in rehabilitation approaches), Tuomi L et al. (2021 [37], heterogeneity in tumor localization/stages), Balaji H et al. (2024 [36], lack of uniformity in cancer sub-sites).

Implications: Variability in treatments, tumor sites, or patient demographics complicates comparisons and may confound results.

### 4.11. Specificity of the Inclusion Criteria

This study did not identify any research meeting the inclusion criteria that investigated antibody-based immunotherapy for relapsed head and neck cancer, despite its recognition as a state-of-the-art treatment in current guidelines [64]. This may reflect the specificity of the inclusion criteria, potentially excluding relevant studies. As highlighted by Zahavi and Weiner (2020) [64], monoclonal antibodies are increasingly critical in cancer therapy, underscoring the need for broader criteria in future reviews to capture such advancements [64].

### 4.12. Overall Impact on the Literature Review

- Reliability: Small sample sizes, missing data, and reliance on self-reported measures introduce variability and potential bias, reducing the confidence in reported outcomes. For example, studies like Williamson A et al. (2021) [2] and Aggarwal P et al. (2023) [4] note wider confidence intervals due to small samples, which weakens the precision of QoL or functional outcome estimates.
- Generalizability: Single-center studies and region-specific protocols (e.g., Bozec A et al., 2022 [27]; Goiato MC et al., 2020 [8]) limit the applicability of findings to diverse populations or healthcare settings. This is particularly relevant for your review if you aim to draw conclusions applicable to global or varied clinical contexts.
- Comparability: Heterogeneity in treatment regimens, patient populations, and study designs (e.g., cross-sectional vs. longitudinal) makes it challenging to synthesize results or perform meta-analyses. For instance, differences in tumor sites (e.g., oropharynx vs. larynx) and treatment modalities (e.g., TORS vs. RT in Scott SI et al., 2023 [11]) complicate direct comparisons.
- Long-Term Insights: Limited follow-up durations and lack of preoperative data (e.g., Larson AR et al., 2021 [28]; Jia L et al., 2025 [41]) restrict the understanding of long-term QoL or functional outcomes, which are critical for head and neck cancer patients given the chronic nature of treatment-related morbidities.
- Clinical Application: Selection bias and subjective measures (e.g., Jimenez JE et al., 2021 [26]; Cardoso RC et al., 2021 [24]) may overestimate or underestimate treatment benefits, potentially misleading clinical decision-making or patient counseling.

## 5. Conclusions

In conclusion, these results underscore the need for a multidisciplinary and personalized approach to head and neck cancer treatment. Integrating de-escalated therapy strategies, careful monitoring of critical functions, and incorporating supportive measures (both physical and psychosocial) are key factors in optimizing oncologic outcomes and patients’ quality of life. Future studies should confirm these findings over the long term and refine prognostic models to provide the most suitable therapeutic solutions for different patient subgroups. We advocate for prospective, randomized designs in order to minimize selection bias and recall bias. We also recommend using objective measures alongside PROMs to reduce reliance on subjective data. This synthesis integrates aspects related to surgical techniques, reconstruction modalities, functional assessment (vocal and swallowing), and supportive interventions, highlighting future research directions and the importance of a holistic approach in managing head and neck cancer patients.

### Implications for Future Research

- Refinement of De-escalation Protocols: Current evidence suggests that de-escalated adjuvant therapy—through reduced radiation doses and adjusted CT regimens—can achieve satisfactory oncologic outcomes while minimizing long-term side effects. Future studies should focus on refining these protocols, evaluating them in larger and more diverse patient groups to confirm safety, efficacy, and sustainability.
- Emphasis on Multidimensional Functional Assessment: Many studies have demonstrated the impact of treatments on swallowing function, voice quality, and HrQoL. Future research must integrate validated PROM alongside objective functional tests (e.g., FEES, V-VST, EP-SHI, HoCoS) to standardize data and enable comparison of results across studies.
- Personalized and Multidisciplinary Approaches: Variability based on factors such as the extent of surgical resection, patient age, socioeconomic status, and psychological stress indicates that a one-size-fits-all strategy is not optimal. Future studies should explore personalized therapies integrating baseline HrQoL assessments, predictive models, and multidisciplinary supportive interventions—including psychological counseling, nutritional support, and dental rehabilitation—to optimize treatment plans tailored to each subgroup.
- Comparative Evaluations of Minimally Invasive Surgical Techniques: Emerging data on robotic-assisted surgery and other minimally invasive techniques suggest significant benefits in functional recovery and complication reduction compared to traditional methods. Future research should conduct direct comparative studies between these new approaches and conventional techniques, emphasizing long-term functional and quality-of-life outcomes.
- Integration of Supportive and Rehabilitation Interventions: The results highlight the critical role of supportive interventions—such as nutritional supplements, vocal rehabilitation programs, and guided self-help exercises—in reducing treatment-associated morbidity. Future research should establish the optimal timing, duration, and combination of these interventions while evaluating their cost-effectiveness and impact on patient recovery.
- Exploration of Dose–Effect Relationships and Toxicity Profiles: A detailed analysis of dose–effect relationships and long-term effects, particularly regarding late toxicities (e.g., trismus, fibrosis, and subclinical swallowing disorders), is necessary. Future studies must investigate the mechanisms underlying these effects and develop strategies or adjuvant therapies to minimize toxicities without compromising tumor control.

In conclusion, these implications emphasize the need for future studies to validate and optimize de-escalation strategies while adopting a holistic approach targeting both oncologic control and comprehensive functional recovery. A multidisciplinary perspective will be essential in designing personalized treatment protocols to significantly improve the QoL for head and neck cancer patients. These research directions open new opportunities to explore mechanisms to counteract treatment toxicities, the benefits of early rehabilitation, and the integration of patient-reported outcomes into clinical practice, which together can lead to better treatment planning and personalization.

This literature review was not registered. There was no protocol prepared for this review.

## Figures and Tables

**Table 1:** Study type and number of patients.

Study Type	NO of Studies	Min.	Max.
Case series	1	3	3
Clinical trial	1	79	79
Cross-sectional study	15	21	892
Nationwide prospective questionnaire-based study	1	203	203
Prospective study	19	28	2171
Randomized controlled trial	8	66	240
Retrospective study	4	21	122
Sub-study from a randomized trial	1	21	21
Survey study	1	33	33

**Table 2:** Summary of treatment modalities for head and neck cancers.

Treatment Category	Description (Authors)
**Surgery**	Selective neck dissection, transoral robotic surgery (TORS), total laryngectomy (TL), partial laryngectomy, free-flap reconstruction, supracricoid partial laryngectomy (SCPL), tracheoesophageal puncture (TEP) (Williamson A, Wulff NB, Bozec A, Jimenez JE, Larson AR, Monte LEFD, Souza FGR, Atula M, Scott SI, D’Andr’ea G, Goiato MC, van Hinte G, Balaji H, Zivkovic A, Liu T, Cocuzza S, Nakai MY)
**Radiotherapy (RT)**	Adjuvant RT, intensity-modulated RT (IMRT), proton therapy (IMPT), dysphagia-optimized IMRT (DO-IMRT), conventional/hyperfractionated RT (Price K, Aggarwal P, Wulff NB, Alvarez-Marcos C, Pingili S, Cardoso RC, Bozec A, Guimaraes I, Balaguer M, Henry M, Scott SI, Korsten LHA, Tuomi L, Rogers SN, Grant SR, Nutting C, Theurer JA, Karlsson T, Johansson M, Li WX)
**Chemotherapy (CT)**	Concurrent chemoradiation (CRT) with cisplatin, docetaxel, or 5-Fluorouracil, induction chemotherapy, targeted therapy (Price K, Aggarwal P, Alvarez-Marcos C, Pingili S, Cardoso RC, Bozec A, Guimaraes I, Balaguer M, Henry M, Yifru TA, Hung CY, Rogers SN, Grant SR, Li WX)
**Combination Therapy**	Surgery with adjuvant RT/CT, chemoradiation, multimodality treatment (surgery, RT, CT) (Pingili S, Guimaraes I, Balaguer M, Yifru TA, Henry M, Korsten LHA, Andreassen R, Scott SI, Deng J, Harrowfield J, Balaji H, Rogers SN, Li WX)
**Novel Interventions**	Nutri-PEITC Jelly, acupuncture with swallowing training, progressive resistance training (PRT), voice rehabilitation, and swallowing exercises (Lam-Ubol A, Zhu X, Hajd’u SF, Karlsson T, Johansson M, Jia L)

Tumor stage, according to TNM classification, was reported in 28 studies: I–II—2; I–III—3; III–IV—15; II–IV—4; II–Iva—1; I–IV—3; IV—2. 21 studies did not explicitly specify the stage.

**Table 3:** PROM INSTRUMENT—number of studies.

	No.
European Organization for Research and Treatment of Cancer QoL Questionnaire-Core 30 (EORTC QLQ-C30)	17
European Organization for Research and Treatment of Cancer—Head and Neck questionnaire (EORTC-H&N35)	14
**Voice-Related QoL questionnaire (V-RQOL)**	2
M D Anderson Dysphagia Inventory (MDADI)	7
Hospital Anxiety and Depression Scale (HADS)	5
Functional Outcome Swallowing Scale (FOSS)	3
Voice Handicap Index-10 (VHI-10)	4
Functional Assessment of Cancer Therapy–General (FACT-G)	4
Consensus Protocol for Auditory–Perceptual Voice Assessment (CAPE-V)	1
Swallowing QoL questionnaire (SWAL-QoL)	4
Supportive Care Needs Survey (SCNS-SF34)	1
Neck Dissection Impairment Index (NDII)	2
Oxford Shoulder Score (OSS)	1
**Mandibular Function Impairment Questionnaire (MFIQ)**	1
Head and Neck Lymphedema and Fibrosis Symptom Inventory (HN-LEF SI)	1
Modified Barium Swallow Study (MBS)	2
Functional Oral Intake Scale (FOIS)	1
Performance Status Scale–Head and Neck (PSS-H&N)	1
European QoL (EQ-5D) Functional Assessment of Cancer Therapy–Head and Neck (FACT-H&N)	3
University of Michigan Xerostomia-related QoL Scale (XeQOLS)	1
Tumor response evaluations Serum p53 and cytochrome c levels (VAG)	2
Progression-free survival (PFS) measurements	1
Patient Concerns Inventory (PCI)	1
Dysphagia Outcome and Severity Scale (DOSS)	1
Speech Handicap Index (SHI)	3
Shoulder Disability Questionnaire (SDQ)	3
Patient Activation Measure (PAM)	2
MD Anderson Symptom Inventory Head and Neck module (MDASI-HN)	2
University of Washington QoL(UW-QOL) score	4
Patient Generated-Subjective Global Assessment (PG-SGA)	1
Patient Health Questionnaire-9 (PHQ-9)	1
Patient Health Questionnaire (PHQ-2)	1
General Anxiety Disorder questionnaire (GAD-2)	1
Shoulder Pain and Disability Index (SPADI) EQ-5D	1
Swedish Self-Evaluation of Communication Experiences After Laryngeal Cancer (S-SECEL)	2
Oral Impact on Daily Performances questionnaire	1
Eating Assessment Tool-10 (EAT-10)	1
Neck Disability Index	2
Dysphagia Handicap Index Kannada (DHI-K)	1
Symptom Check List	2
EORTC QLQ H&N43	2
Three-Item Loneliness Scale	1
Beck Depression Inventory (BDI)	2

**Table 4:** Main result of the studies.

Authors, Year, Region	No.	Evaluation Methods	Main Results
Williamson A et al., 2021 United Kingdom	3	UW-QOL MDADI	Histopathological analysis verified complete removal of the primary tumor in all instances. Two patients had smooth recoveries, but one experienced a chest infection and tracheocutaneous fistula, treated non-surgically. The average hospital stay was 15 days [2].
Price K et al., 2022 United States	79	MBS FOIS PSS-H&N EQ-5D FACT-H&N EORTC QLQ-H&N35 XeQOLS	Low rates of long-term toxic effects. Improved swallowing function by 12 months post-treatment; QoL returned to baseline levels over time. No patients required long-term feeding tube dependence [3].
Aggarwal P et al., 2023 United States	880	MDASI-HN	In total, 64.4% of survivors reported mild to severe hearing symptoms. Hearing loss and tinnitus were significantly associated with worse HrQoL. Moderate to severe hearing loss and tinnitus increased the odds of reporting moderate to severe symptom distress [4].
Wulff NB et al., 2022 Denmark and Sweden	172	EORTC QLQ-C30, EORTC QLQ-H&N35, V-RQOL MDADI, HADS	Participants scored worse than normative reference populations on nearly all scales of the EORTC questionnaires. A total of 46% experienced moderate/severe dysphagia, 57% had moderate/severe voice problems, 16% had depression, and 20% had anxiety. Increasing voice problems, dysphagia, depression, and comorbidities were associated with lower HrQoL [5].
Álvarez-Marcos C et al., 2022 Spain	21	EORTC-H&N35, VHI, CAPE-V	Voice changes were frequent, with alterations in all CAPE-V attributes. A total of 78% of patients showed type II and III spectrograms in acoustic analysis. EORTC-H&N35 scores showed a reduction in 10–40% of items related to voice [10].
Pingili S et al., 2021 India	97	EORTC QOL-H&N35, MFIQ	Most commonly reported symptoms: xerostomia (93.81%), pain (81.44%), dysphagia (76.3%). In total, 40.2% of patients experienced malnutrition. Malnutrition was lower in patients who had nutritional supplements. QoL deteriorated immediately after treatment but improved over time [19].
Cardoso RC et al., 2021 United States	892	MDASI-HN. EQ-5D EuroQol-5D MDADI	In total, 31% of patients reported trismus. Severity of trismus negatively impacted QoL. Trismus correlated with increased dysphagia and dietary restrictions. Patients with severe trismus were more likely to be feeding tube-dependent. Adherence to jaw stretching exercises was associated with lower trismus prevalence [24].
Bozec A et al., 2020 France	64	EORTC QLQ-C30, QLQ-H&N35, QLQ-ELD14. HADS. PCI DOSS	Long-term QoL and functional measures remained largely intact. Primary ongoing issues included fatigue, constipation, and oral function difficulties. Salivary dysfunction and challenges with chewing/swallowing were key patient concerns. Significant psychological distress (HADS score ≥ 15) and frailty (G8 score < 15) correlated strongly with reduced QoL [27].
Guimarães I et al., 2021 Portugal	95	EP-SHI	The EP-SHI demonstrated strong reliability and validity, distinguishing between patients and healthy individuals. It showed significant correlations with the European Portuguese Voice Handicap Index (EP-VHI) [14].
Balaguer M et al., 2023 France	25	ECVB, DIP, PHI, CHI, EORTC QLQ-C30, EORTC QLQ-H&N35	A holistic communication score (HoCoS) was developed to measure the impact of speech disorders on communication in patients treated for oral or oropharyngeal cancer. The score showed good reliability (rs = 0.91) and validity [15].
Jimenez JE et al., 2021 United States	80	EAT-10, UW-QOL NDI, PHQ-2, GAD-2	The degree of tongue resection was closely linked to diminished quality-of-life outcomes, particularly in patients with oral tongue defects [26].
Larson AR et al., 2021 United States	24	UW-QOL 4.	Acceptable QoL outcomes in swallowing and speech; anterior floor of mouth (A-FOM) patients reported worse chewing outcomes compared to lateral floor of mouth (L-FOM) patients [28].
Monte LEFD et al., 2024 Brazil	31	UW-QOL.	Significant improvements were observed in domains like speech, pain, appearance, activity, recreation, mood, and anxiety. However, no statistical significance was found for swallowing, chewing, taste, and saliva [29].
Souza FGR et al., 2020 Brazil	95	UW-QOL, FACT-HN, EORTC QLQ-H&N35.	Patients using tracheoesophageal prostheses reported superior QoL compared to those relying on electrolarynx or esophageal speech. Lack of vocal output was tied to poorer quality of life [30].
Andreassen R et al., 2022 Norway	216	Oral Impact on Daily Performances questionnaire	Survivors of head and neck cancer faced a fourfold higher likelihood of reporting issues with daily activities compared to the general population. Eating and food enjoyment were the most commonly impacted areas [31].
Yifru TA et al., 2021 Ethiopia	102	MDADI	The composite mean MDADI score was 53.29, reflecting impaired swallowing-related QoL. Factors such as female gender, low income, advanced tumor stage, and laryngeal cancer were associated with poorer QoL [9].
Atula M 2024 Finland	203	SSQ BDI, Three-Item Loneliness Scale.	No association was found between psychosocial factors and patient delay. Patients with large head and neck cancers had lower socioeconomic status and higher depression rates compared to the general Finnish population [18].
Hung CY 2024 Taiwan	461	EORTC QLQ-HN35	Patients with higher QLQ-HN35 scores had an increased risk of incomplete CCRT (13.4% vs. 6.5%, OR = 2.22, *p* = 0.015). Higher scores were associated with more emergency room visits (36.4% vs. 27.0%, OR = 1.55, *p* = 0.030) and unexpected hospitalizations (33.8% vs. 19.6%, OR = 2.10, *p* = 0.001). Higher scores correlated with increased grade 3 hematological (34.2% vs. 21.3%, OR = 1.92, *p* = 0.002) and non-hematological toxicities (78.8% vs. 68.7%, OR = 1.69, *p* = 0.014). Lower QLQ-HN35 scores were linked to better overall survival (OS) and disease-free survival (DFS) [6].
Henry M et al., 2022 Canada	146	HADS, FACT-G + Head and Neck Module, SCNS-SF34	HPV-negative patients had higher anxiety and depression levels immediately post-diagnosis. HPV-positive patients showed lower psychological distress but had increased vulnerability post-treatment. Major depressive disorder (MDD) significantly impacted anxiety, depression, and QoL in HPV-positive patients [16].
Scott SI et al., 2021 United States	44	EORTC QLQ-C30, EORTC QLQ-HN35 MDADI, NDII, OSS	Salivary flow rates dropped significantly in the RT group at 12 months. The RT group also showed the largest declines in QoL scores related to dry mouth and sticky saliva. Swallowing function worsened in both groups at 12 months, while shoulder impairment was uncommon in both [32].
D’Andréa G et al., 2022 France	53	MDADI, EORTC QLQ-C30, EORTC QLQ-H&N35	MDADI total scores at preoperative, 1-year, and 2-year marks were 71.4, 64.3, and 57.5, respectively. QLQ-C30 global scores at the same intervals were 61.2, 59.4, and 80.6. Decannulation was achieved in 97.1% of tracheotomized patients. Two-year enteral tube dependency was 23.1%. Two-year overall survival, disease-free survival, and local control rates were 59%, 46.1%, and 80.9%, respectively [21].
Andersen LP et al., 2023 United States	115	EORTC QLQ-C30, HADS, BHLS	Median global HrQoL score was 67.7 (IQR = 50.0, 83.4). Anxiety and depression were significantly inversely correlated with QoL. Higher income and early-stage cancer were associated with better physical functioning [17].
Korsten LHA et al., 2021 Netherlands	270	(EORTC QLQ-C30) (EORTC QLQ-HN35)	Patients with HPV-positive tumors had better QoL before treatment, worsened more during treatment, but recovered better and faster at follow-up. Differences in global quality of life, physical, role, and social functioning, fatigue, pain, insomnia, and appetite loss were observed between HPV-positive and HPV-negative patients. Oral pain followed a different trajectory, with HPV-positive patients reporting lower pain before treatment and at certain follow-up points [7].
Goiato MC et al., 2020 Brazil	50	EORTC QLQ-C30 EORTC QLQ-HN35	QoL was significantly affected by treatment type and tumor location. Patients treated with surgery plus RT had worse QoL scores compared to those treated with surgery alone. The period of greatest morbidity was 1 week after treatment. QoL scores improved over time, with many returning to baseline levels after 3 months [8].
Scott SI et al., 2023 Denmark	44	EORTC QLQ-C30, EORTC QLQ-HN35, MDADI	Significant improvement in swallowing function from 1 to 3 years post-treatment. The TORS group showed better safety scores and swallowing efficiency. QoL improvements were noted only in TORS-treated patients. The RT group showed deterioration in QoL scores despite functional improvements [32].
Deng J et al., 2022 United States	117	HN-LEF SI, VHNSS, EORTC QLQ-C30 HADS, SF-MPQ, NDI.	The study validated the HN-LEF SI by demonstrating expected correlations with existing quality-of-life and symptom burden measures, confirming its construct validity [33].
Harrowfield J et al., 2021 Australia	83	PG-SGA, PHQ-9, EORTC QLQ-C30.	HPV-positive patients were more likely to experience > 10% weight loss three months post-treatment. No notable difference in malnutrition rates was observed between HPV-positive and HPV-negative patients during treatment [34].
Ramalingam K et al., 2024 India	111	EORTC QLQ-C30 and QLQ-HN43.	Light gradient boosting predicted cancer patients’ QoL with 96% accuracy and 0.20 log loss [35].
van Hinte G et al., 2021 Netherlands	69	SDQ, SPADI, EQ-5D, EORTC-QLQ-HN35.	SLNB patients had better short-term shoulder function compared to END patients; no significant differences in long-term health-related quality of life [25].
Balaji H et al., 2024 India	53	SHI-K DHI-K.	Poor agreement between clinician-rated and patient-reported outcomes for speech and swallowing (ICC values: 0.480 for speech, 0.471 for swallowing) [36].
Tuomi L et al., 2021 Sweden	28	EORTC QLQ-C30, EORTC QLQ-H&N35, S-SECEL	No significant changes in HrQoL perceptual voice quality over 24 months post-RT. However, HrQoL scores remained inferior to those of healthy controls, with significant deterioration in dry mouth and sticky saliva [37].
Rogers SN et al., 2020 United Kingdom	2171	EORTC QLQ-C30	Higher baseline HrQoL scores were associated with improved survival rates across most tumor sites. Specific functional domains like physical, role, and social functioning showed significant associations with survival [38].
Zivkovic A et al., 2024 Serbia	56	EORTC QLQ-H&N43	Significant predictors of QoL included T stage, pain intensity, and extent of surgical procedure. Oxidative stress markers (SOD, MDA) were linked to postoperative complications [39].
Grant SR et al., 2020 United States	71	MDADI	Swallowing function declined sharply during treatment but showed rapid recovery by 10 weeks post-treatment, with steady improvement through two years [40].
Jia L et al., 2025 China.	30	MDADI, FEES, VFSS.	FEES showed high sensitivity (84%) and specificity (94%) for detecting aspiration. MDADI scores indicated significant swallowing difficulties post-surgery, with improvements over time [41].
Nutting C et al., 2023 United Kingdom and Ireland	112	MDADI	Patients in the DO-IMRT group had significantly higher MDADI composite scores at 12 months compared to the standard IMRT group (mean score 77.7 vs. 70.6, *p* = 0.037). DO-IMRT led to lower radiation doses to the pharyngeal constrictor muscles. Serious adverse events were reported in 23 patients, with common late adverse events, including hearing impairment, dry mouth, and dysphagia [23].
Lam-Ubol A et al., 2023 Thailand	72	HrQoL assessments, PFS measurements, Tumor response evaluations, Serum p53 and cytochrome c levels	The study group showed improved HrQoL and stable disease compared to the control group. Progression-free survival was significantly longer in the study group. Serum p53 levels increased in the study group, suggesting potential p53 reactivation. No serious intervention-related adverse events occurred [20].
Aggarwal P et al., 2021 Netherlands	92	SWAL-QOL. SHI SDQ. EORTC QLQ-C30 & QLQ-H&N35 PAM	Patients in the intervention group reported fewer swallowing and communication problems over time. No significant differences were found in speech, shoulder problems, patient activation, or overall quality of life. Patients within 6 months post-surgery benefited most from the intervention [42].
Hajdú SF et al., 2022 Denmark	240	EORTC QLQ C-30, EORTC QLQ-H&N35, MD MDADI NRS. MDI SCL-92 Anxiety subscale.	Significant improvements in mouth opening, QoL, depression, and anxiety at 12 months in the intervention group compared to non-active controls. No significant effect on swallowing safety [43].
Zhu X et al., 2022 China	71	VFSE MDADI, QLQ-C30	The experimental group showed significantly higher effective rates (97.1%) and complete remission rates (36.1%) compared to the control group (60% and 14.3%, respectively). Improvements in VFSE, MDADI, and QLQ-C30 scores were significantly greater in the experimental group [44].
Karlsson T et al., 2022 Sweden	74	S-SECEL, GRBAS protocol grade, Roughness, Breathiness, Asthenia, Strain, Acoustic analysis.	The intervention group showed significant improvements in communication experiences and perceptual voice qualities (breathiness and strain) over three years. The control group demonstrated deterioration in roughness [45].
Jansen F et al., 2020 Netherlands.	92	SWAL-QOL SHI, SDQ, EORTC QLQ-C30/H&N35, PAM	The intervention group showed progress in swallowing and communication issues over time compared to the control group. No significant differences were noted for speech, shoulder issues, patient activation, or HrQoL [46].
Johansson M et al., 2020 Sweden	66	EORTC QLQ-C30 mapped to EQ-5D values for quality-adjusted life years QALYs.	Voice rehabilitation improved HrQoL and communicative function, preventing deterioration of voice quality over time [47].
Li WX et al., 2023 China	64	FOSS, VHI-10 FACT-G	Three-year OS was 60.7%, and five-year OS was 47.3%. Patients with Stage I or II disease had significantly higher OS than those with Stage III or IV. Decannulation succeeded in 85.9% of patients, and 78.1% achieved satisfactory swallowing function. The median FACT-G quality-of-life score was 75 [22].
Li WX et al., 2022 China	122	FOSS, FACT-G	Five-year OS and disease-free survival (DFS) were 40.0% and 36.1%, respectively. Swallowing function was satisfactory in 73.8% of patients. Tracheostomy-free survival was achieved in 45.1% of patients. Local–regional recurrence and distant metastasis were independent impact factors for OS and DFS [48].
Liu T et al., 2024 China	21	MDADI VHI-10.	Patients showed satisfactory recovery in swallowing and voice function. The mean MDADI score was 92.67, indicating good swallowing-related quality of life. The mean VHI-10 score was 7.14, reflecting minimal impact of voice disorders on QoL [12].
Cocuzza S et al., 2020 Italy	54	V-RQoL VHI.	Tracheoesophageal voice prosthesis showed better socio-emotional and functional outcomes compared to esophageal speech. However, fistula-related complications negatively impacted quality of life [13].
Theurer JA et al., 2025 Canada	21	MDADI, MBSImP, PAS.	Swallowing profiles were not significantly different between treatment arms. Pharyngeal swallowing impairments were weakly associated with MDADI subscales and PAS scores [49].
Nakai MY et al., 2021 Brazil	33	EORTC QLQ-C30 and H&N35	SPL patients scored better in global health status–QoL and general activities, with fewer sensory and speech-related symptoms compared to TL patients [50].

**Table 5:** Main conclusion and limitations.

Authors, Year, Region	Tumor Site	Main Conclusion	Study Limits
Williamson A et al., 2021 United Kingdom	Oropharynx	ORS-assisted resection combined with RFFF reconstruction can achieve good oncological and quality-of-life outcomes with acceptable postoperative complications [2].	Small sample size, limited follow-up duration, and lack of direct comparison with conventional surgical approaches.
Price K et al., 2022 United States	Oropharynx	De-escalated adjuvant therapy resulted in excellent swallow outcomes and preserved QoL. Lower radiation doses reduced long-term toxic effects. Further studies are needed to confirm long-term benefits [3].	Single-institution study. Limited generalizability due to specific inclusion criteria. Potential selection bias due to exclusion criteria.
Aggarwal P et al., 2023 United States	Oropharynx	The research emphasizes the importance of ongoing audiological assessments and monitoring to identify hearing issues early. Prompt intervention may reduce the long-term effects on quality of life [4].	Small sample sizes led to wider confidence intervals. Variability in treatment regimens and patient selection may have influenced results.
Wulff NB et al., 2022 Denmark and Sweden	Hypopharynx and Larynx	A substantial proportion of patients experienced clinically significant late effects, which negatively impacted HrQoL. Voice problems, dysphagia, depression, and anxiety were independently associated with lower HrQoL [5].	Single-center study, limiting generalizability. Variability in rehabilitation approaches across regions.
Álvarez-Marcos C et al., 2022 Spain	Hypopharynx and Larynx	Subclinical voice disorders are common after chemo-RT. Although patients consider vocal impairment minor, it may contribute to reduced quality of life [10].	Small sample size. Single-center study, limiting generalizability.
Pingili S et al., 2021 India	Oral and Oropharynx	Treatment significantly impacts quality of life, but recovery improves symptoms over time. Nutritional supplements play a crucial role in reducing malnutrition [19].	Single-center study, limiting generalizability. Modified questionnaires to suit the Indian population. Small sample size.
Cardoso RC et al., 2021 United States	Oral and Oropharynx	Trismus is a prevalent and impactful morbidity in long-term oropharyngeal cancer survivorship. Advanced disease stages, tumor sub-site (tonsil), and CCT were associated with increased trismus prevalence. Further investigation is needed to explore dose–effect relationships on muscles of mastication [24].	Self-reported trismus assessment may introduce bias. Limited generalizability due to single-institution study. Potential selection bias due to exclusion criteria. Small sample size for certain subgroups (IMPT and PORT patients).
Bozec A et al., 2022 France	Oropharynx	An inverse relationship was observed between patient concerns and quality of life. Dental restoration, psychological care, and nutritional support are essential for managing elderly OOPC patients [27].	Limited generalizability due to France-specific treatment protocols. Small sample size. Potential selection bias due to exclusion criteria.
Guimaraes I et al., 2021 Portugal	Oral and Oropharynx	The EP-SHI is a culturally relevant, valid, and reliable PROM for assessing speech-related QoL in oral and oropharyngeal cancer patients [14].	Modest sample size, lack of objective speech measures, and limited representation of oropharyngeal cancer patients.
Balaguer M et al., 2023 France	Oropharynx	The HoCoS fills a gap in head and neck oncology by providing a comprehensive measure of communication impairments. It allows for a better understanding of functional and psychosocial consequences in patient follow-up [15].	Small sample size; requires further validation on a larger cohort.
Jimenez JE et al., 2021 United States	Lips and Oral Cavity	The extent of tongue resection was strongly associated with poor QoL outcomes after free tissue reconstruction of the oral cavity. This factor mediates the associations between other defect characteristics and QoL. The findings emphasize the importance of considering expected oral tongue defects when counseling patients and highlight the need for a multidisciplinary approach to postoperative care [26].	The subjective nature of PROM, the retrospective cross-sectional design, and the variability in the time elapsed since treatment. Additionally, the sample consisted mostly of white patients, highlighting disparities in access to survivorship services. The study also lacked pre-treatment comparisons and had potential sampling bias, as patients attending survivorship clinics may differ from those who do not.
Larson AR et al., 2021 United States	Lips and Oral Cavity	STSG reconstruction is a reasonable option for early-stage floor of mouth carcinoma, though A-FOM may benefit from alternative reconstruction methods [28].	Small sample size, low questionnaire response rate, lack of preoperative functional data, and absence of tumor depth information.
Monte LEFD et al., 2024 Brazil	Larynx	The electrolarynx is a viable and effective method for voice rehabilitation, positively impacting the QoL of laryngectomy patients [29].	Small sample size, cross-sectional design, and limited generalizability due to the specific patient population.
Souza FGR et al., 2020 Brazil	Larynx	Tracheoesophageal prosthesis (TEP) is the gold standard for vocal rehabilitation, providing better QoL for TL patients [30].	Recall bias due to long intervals since surgery, cross-sectional design limiting generalizability, and potential underrepresentation of the broader patient population.
Andreassen R et al., 2022 Norway	Head and Neck	Head and neck cancer treatment is associated with lasting impairment of oral HrQoL. A multidisciplinary approach and access to expert dental care are recommended to improve OHrQoL [31].	Cross-sectional design limits causal inferences; potential recall bias due to self-reported data.
Yifru TA et al., 2021 Ethiopia	Head and Neck	Swallowing-related QoL is significantly impacted by dysphagia in head and neck cancer patients. Incorporating swallowing assessments into treatment protocols is recommended [9].	Cross-sectional design limits causal inferences; potential recall bias due to self-reported data; findings may not be generalizable beyond the Ethiopian context.
Atula M 2024 Finland	Head and Neck	Psychosocial factors did not influence patient delay, but socioeconomic status and depression should be considered in clinical practice [18].	A large number of patients were excluded or unable to participate, potential for recall bias, and the inability to assess psychological status prior to cancer diagnosis.
Hung CY 2024 Taiwan	Head and Neck	Pre-treatment HrQoL significantly impacts treatment-related complications, tolerance, and survival outcomes. QLQ-HN35 is a valuable predictor for treatment tolerance and outcomes in head and neck cancer patients [6].	Single-center study, limiting generalizability. HrQoL was only assessed at baseline, without follow-up evaluations. Factors influencing HrQoL, such as socioeconomic status, were not fully explored.
Henry M et al., 2022 Canada	Head and Neck	HPV-negative patients generally experience greater psychological distress at diagnosis. HPV-positive patients require equal psychological support post-treatment. Head and neck clinics should address MDD, anxiety, depression, and quality of relationships [16].	Single-center study, limiting generalizability. The majority of participants were male. Some missing data required imputation.
Scott SI et al., 2021 United States	Oropharynx	Functional and QoL outcomes were generally positive 1 year after treatment. Persistent impairment was observed in both groups, particularly in swallowing function [32].	Small sample size. Limited generalizability due to a single-center study.
D’Andréa G et al., 2022 France	Oropharynx	Robotic-assisted salvage surgery demonstrated satisfactory quality of life, good functional sequelae, and favorable oncological outcomes compared to historical approaches [21].	Single-center study, limiting generalizability. Small sample size. A high proportion of HPV-negative patients, which may affect outcomes.
Andersen LP et al., 2023 United States	Oropharynx	Patients with lower income, advanced cancer stage, and anxiety/depression had poorer QoL. Screening for these factors before treatment could improve patient support and outcomes [17].	Conducted in a single U.S. region, limiting generalizability. The majority of participants were white and male. Some missing data required imputation.
Korsten LHA et al., 2021 Netherlands	Oropharynx	HPV-positive patients generally recover better in terms of QoL compared to HPV-negative patients. Findings highlight the importance of tailoring supportive care based on HPV status [7].	Limited generalizability due to Dutch-specific treatment protocols. Potential selection bias due to exclusion criteria. Missing surveys due to patient death or loss to follow-up.
Goiato MC et al., 2020 Brazil	Oral and Oropharynx	QoL is significantly impacted by oral and oropharyngeal cancer treatment. Patients treated with surgery plus RT experience greater morbidity. Short-term follow-up is crucial for understanding recovery trends [8].	Small sample size. Short follow-up period (only 3 months). Limited generalizability due to Brazil-specific treatment protocols.
Scott SI et al., 2023 Denmark	Oropharynx	TORS patients demonstrated better long-term swallowing function and QoL. RT patients showed functional recovery but persistent QoL decline. Further studies are needed to assess long-term recovery trends [32].	Small sample size, particularly in the RT group. TORS and RT groups are not directly comparable due to different eligibility criteria. Limited generalizability due to Denmark-specific treatment protocols.
Deng J et al., 2022 United States	Oropharynx	The HN-LEF SI is a reliable and valid patient-reported outcome measure for assessing symptom burden and functional impairment due to lymphedema and fibrosis in head and neck cancer patients [33].	Limited diversity in patient demographics, single-institution study, and potential sampling bias.
Harrowfield J et al., 2021 Australia	Oropharynx	Both HPV-positive and HPV-negative OPSCC patients experience nutritional decline during treatment, requiring equally intense nutritional intervention. HPV-positive patients may need additional support during recovery [34].	Small sample size, lack of long-term follow-up, and limited geographic diversity.
Ramalingam K et al., 2024 India	Lips and Oral Cavity	The prediction model can help oral surgeons and oncologists improve planning and therapy for oral cancer patients [35].	Modest sample size, reliance on patient-reported outcomes, and variability in time elapsed since treatment.
van Hinte G et al., 2021 Netherlands	Lips and Oral Cavity	SLNB is a preferred treatment strategy due to better short-term shoulder function and cost-effectiveness [25].	Small sample size, lack of accessory nerve status data, and missing physiotherapy treatment details.
Balaji H et al., 2024 India	Lips and Oral Cavity	No agreement between patient-reported and clinician-rated outcomes; PROMs should be incorporated into routine clinical practice for comprehensive care [36].	Modest sample size, lack of uniformity in cancer sub-sites, reliance on caregivers for illiterate participants, and absence of socioeconomic data.
Tuomi L et al., 2021 Sweden	Hypopharynx and Larynx	Patients with laryngeal cancer may require support in areas such as nutrition, swallowing, and voice rehabilitation up to 24 months post-RT [37].	Small sample size, high dropout rate, and heterogeneity in tumor localization and stages.
Rogers SN et al., 2020 United Kingdom	Head and Neck	Baseline HRQOL is a valuable prognostic indicator for survival in head and neck cancer patients. Incorporating HRQOL into routine clinical care can enhance patient–clinician decision-making and recovery [38].	Limited generalizability due to UK-based data, underrepresentation of older patients and those with poorer HRQOL, and incomplete data from some participants.
Zivkovic A et al., 2024 Serbia	Larynx	Extensive surgery and complications increase oxidative stress and inflammation, impacting QoL. Radical procedures correlate with higher symptom burden [39].	Small sample size, single-center study, and lack of preoperative psychological assessments.
Grant SR et al., 2020 United States	Oropharynx	IMPT does not confer additional excess toxicity related to swallowing compared to photon-based RT [40].	Single-institution study, decreasing patient numbers over long-term follow-up, and reliance on MDADI as the sole swallowing function measure.
Jia L et al., 2025 China.	Larynx	Dysphagia significantly impacts early QoL post-laryngectomy. FEES is effective for early swallowing function evaluation and rehabilitation guidance [41].	Small sample size, limited follow-up duration, and exclusion of severely malnourished or non-compliant patients.
Nutting C et al., 2023 United Kingdom and Ireland	Oropharynx and Hypopharynx	DO-IMRT improved patient-reported swallowing function compared to standard IMRT. It should be considered a new standard of care for pharyngeal cancer RT [23].	Single-center study limits generalizability. The majority of participants were male. Missing data required imputation.
Lam-Ubol A et al., 2023 Thailand	Oropharynx	Nutri-PEITC Jelly intake for 3 months is safe and improves QoL and PFS. Potential for PEITC to stabilize disease progression in advanced oral and oropharyngeal cancer. Further studies are needed to confirm long-term effects and mechanisms [20].	Small sample size. Limited generalizability due to Thailand-specific treatment protocols. Potential selection bias due to exclusion criteria.
Aggarwal P et al., 2021 Netherlands	Oropharynx	The guided self-help exercise program improves swallowing and communication. Time since treatment influences effectiveness, with early intervention showing better results. Further research is needed to optimize rehabilitation strategies [42].	Moderate adherence to the exercise program (59%). Limited generalizability due to Dutch-specific treatment protocols. Small sample size, particularly in subgroups.
Hajdú SF et al., 2022 Denmark	Head and Neck	The intervention showed benefits for secondary outcomes like QoL and mental health, but did not improve swallowing safety. Longer intervention durations and continued rehabilitation may be needed to mitigate functional deterioration in HNC survivors [43].	The intervention period may have been too short; differences between groups were relatively small; high dropout rate (25% at 12 months).
Zhu X et al., 2022 China	Larynx	Combining acupuncture with swallowing exercises significantly enhances swallowing ability and QoLin post-surgical laryngeal cancer patients with dysphagia [44].	Small sample size, single-center study, and lack of long-term follow-up data.
Karlsson T et al., 2022 Sweden	Larynx	Voice rehabilitation following RT for laryngeal cancer has long-term positive effects on communication and voice quality [45].	Reduced number of participants over time, baseline differences between groups, and lack of significant acoustic findings.
Jansen F et al., 2020 Netherlands.	Larynx	The guided self-help exercise program effectively improves swallowing and communication in TL patients [46].	Low adherence rate (59%) to the exercise program and lack of significant effects on shoulder problems, self-management, and HRQoL.
Johansson M et al., 2020 Sweden	Larynx	Voice rehabilitation following RT for laryngeal cancer is cost-saving from a societal perspective and provides better health outcomes [47].	Small sample size, large variation in healthcare utilization and production loss, and potential recall bias in reporting sick leave days.
Li WX et al., 2023 China	Hypopharynx and Larynx	LPS and MAT provide satisfactory oncologic control and good functional outcomes for selected patients, especially those with early-stage disease [22].	Retrospective design. Single-center study, limiting generalizability. High proportion of male patients.
Li WX et al., 2022 China	Hypopharynx and Larynx	Comprehensive treatment centered on surgery can achieve effective swallowing function while maintaining oncological control. Surgical defect size, local–regional recurrence, and distant metastasis independently affected survival. Pharyngo-cutaneous fistula and local–regional recurrence independently influenced swallowing function. Clinical stage, local–regional recurrence, decannulation, and feeding tube independently impacted quality of life [48].	Single-center study, limiting generalizability. Retrospective design.
Liu T et al., 2024 China	Larynx	SCPL is effective in preserving laryngeal function while ensuring oncological safety, making it a viable surgical option for laryngeal cancer [12].	Small sample size, single-center study, and potential bias due to the exclusion of patients with severe complications or incomplete follow-up.
Cocuzza S et al., 2020 Italy	Larynx	TEP is effective for voice rehabilitation, but complications like fistula-related disorders require careful management to optimize quality of life [13].	Modest sample size, non-randomized design, and potential bias due to non-standardized protocols.
Theurer JA et al., 2025 Canada	Oropharynx	Instrumental swallowing assessments should be strongly considered alongside quality-of-life measures to best describe swallowing outcomes in studies of RT and/or surgery [49].	Small sample size, limited follow-up duration, and lack of direct comparison with conventional surgical approaches.
Nakai MY et al., 2021 Brazil	Larynx	SPL is associated with better QoL than TL and should be considered for advanced laryngeal cancer treatment despite swallowing rehabilitation challenges [50].	Small sample size, single-center study, and potential bias due to missing data and lack of preoperative QoL assessments.

## Data Availability

The data presented in this study are available in this article.

## References

[1] Page M.J., McKenzie J.E., Bossuyt P.M., Boutron I., Hoffmann T.C., Mulrow C.D., Shamseer L., Tetzlaff J.M., Akl E.A., Brennan S.E. (2021). The PRISMA 2020 statement: An updated guideline for reporting systematic reviews. BMJ.

[2] Williamson A., Haywood M., Awad Z. (2021). Feasibility of Free Flap Reconstruction Following Salvage Robotic-Assisted Resection of Recurrent and Residual Oropharyngeal Cancer in 3 Patients. Ear Nose Throat J..

[3] Price K., Van Abel K.M., Moore E.J., Patel S.H., Hinni M.L., Chintakuntlawar A.V., Graner D., Neben-Wittich M., Garces Y.I., Price D.L. (2022). Long-Term Toxic Effects, Swallow Function, and Quality of Life on MC1273: A Phase 2 Study of Dose De-escalation for Adjuvant Chemoradiation in Human Papillomavirus-Positive Oropharyngeal Cancer. Int. J. Radiat. Oncol. Biol. Phys..

[4] Aggarwal P., Nader M.E., Gidley P.W., Pratihar R., Jivani S., Garden A.S., Mott F.E., Goepfert R.P., Ogboe C.W., Charles C. (2023). Association of hearing loss and tinnitus symptoms with health-related quality of life among long-term oropharyngeal cancer survivors. Cancer Med..

[5] Wulff N.B., Dalton S.O., Wessel I., Arenaz Búa B., Löfhede H., Hammerlid E., Kjaer T.K., Godballe C., Kjaergaard T., Homøe P. (2022). Health-Related Quality of Life, Dysphagia, Voice Problems, Depression, and Anxiety After Total Laryngectomy. Laryngoscope.

[6] Hung C.Y., Hsu M.H., Lee S.H., Hsueh S.W., Lu C.H., Yeh K.Y., Wang H.M., Chang J.T., Hung Y.S., Chou W.C. (2024). Impact of pretreatment quality of life on tolerance and survival outcome in head and neck cancer patients undergoing definitive CCRT. J. Formos. Med. Assoc..

[7] Korsten L.H.A., Jansen F., Lissenberg-Witte B.I., Vergeer M., Brakenhoff R.H., Leemans C.R., Verdonck-de Leeuw I.M. (2021). The course of health-related quality of life from diagnosis to two years follow-up in patients with oropharyngeal cancer: Does HPV status matter?. Support. Care Cancer.

[8] Goiato M.C., Amoroso A.P., Silva B., Dos Santos E.G., Caxias F.P., Bitencourt S.B., Moreno A., Dos Santos D.M. (2020). The Impact of Surgery and Radiotherapy on Health-Related Quality of Life of Individuals with Oral and Oropharyngeal Carcinoma and Short-Term Follow up after Treatment. Asian Pac. J. Cancer Prev..

[9] Yifru T.A., Kisa S., Dinegde N.G., Atnafu N.T. (2021). Dysphagia and its impact on the quality of life of head and neck cancer patients: Institution-based cross-sectional study. BMC Res. Notes.

[10] Álvarez-Marcos C., Vicente-Benito A., Gayol-Fernández Á., Pedregal-Mallo D., Sirgo-Rodríguez P., Santamarina-Rabanal L., Llorente J.L., López F., Rodrigo J.P. (2022). Voice outcomes in patients with advanced laryngeal and hypopharyngeal cancer treated with chemo-radiotherapy. Acta Otorhinolaryngol. Ital..

[11] Scott S.I., Madsen A.K.Ø., Rubek N., Charabi B.W., Wessel I., Jensen C.V., Friborg J., von Buchwald C. (2023). Dysphagia and QoL 3 Years After Treatment of Oropharyngeal Cancer With TORS or Radiotherapy. Laryngoscope.

[12] Liu T., Feng H., Liang Z., Xu S., Qin G. (2024). Analysis of swallowing and voice-related quality of life in patients after supracricoid partial laryngectomy. Eur. Arch. Otorhinolaryngol..

[13] Cocuzza S., Maniaci A., Grillo C., Ferlito S., Spinato G., Coco S., Merlino F., Stilo G., Santoro G.P., Iannella G. (2020). Voice-Related Quality of Life in Post-Laryngectomy Rehabilitation: Tracheoesophageal Fistula’s Wellness. Int. J. Environ. Res. Public Health.

[14] Guimarães I., Sousa A.R., Gonçalves M.F. (2021). Speech handicap index: Cross-cultural adaptation and validation in European Portuguese speakers with oral and oropharyngeal cancer. Logop. Phoniatr. Vocol..

[15] Balaguer M., Pinquier J., Farinas J., Woisard V. (2023). Development of a holistic communication score (HoCoS) in patients treated for oral or oropharyngeal cancer: Preliminary validation. Int. J. Lang. Commun. Disord..

[16] Henry M., Arnovitz E., Frenkiel S., Hier M., Zeitouni A., Kost K., Mlynarek A., Black M., MacDonald C., Richardson K. (2022). Psychosocial outcomes of human papillomavirus (HPV)- and non-HPV-related head and neck cancers: A longitudinal study. Psychooncology.

[17] Andersen L.P., Dietrich M.S., Murphy B.A., Deng J. (2023). Factors associated with quality of life among patients with a newly diagnosed oral cavity and oropharyngeal cancer. Eur. J. Oncol. Nurs..

[18] Atula M., Atula T., Aro K., Irjala H., Halme E., Jouppila-Mättö A., Koivunen P., Wilkman T., Mäkitie A., Elovainio M. (2024). Psychosocial factors and patient and healthcare delays in large (class T3-T4) oral, oropharyngeal, and laryngeal carcinomas. BMC Cancer.

[19] Pingili S., Ahmed J., Sujir N., Shenoy N., Ongole R. (2021). Evaluation of Malnutrition and Quality of Life in Patients Treated for Oral and Oropharyngeal Cancer. Sci. World J..

[20] Lam-Ubol A., Sukhaboon J., Rasio W., Tupwongse P., Tangshewinsirikul T., Trachootham D. (2023). Nutri-PEITC Jelly Significantly Improves Progression-Free Survival and Quality of Life in Patients with Advanced Oral and Oropharyngeal Cancer: A Blinded Randomized Placebo-Controlled Trial. Int. J. Mol. Sci..

[21] D’Andréa G., Bordenave L., Nguyen F., Tao Y., Paleri V., Temam S., Moya-Plana A., Gorphe P. (2022). A prospective longitudinal study of quality of life in robotic-assisted salvage surgery for oropharyngeal cancer. Eur. J. Surg. Oncol..

[22] Li W.X., Dong Y.B., Lu C., Bradley P.J., Liu L.F. (2023). Efficacy of Larynx Preservation Surgery and Multimodal Adjuvant Therapy for Hypopharyngeal Cancer: A Case Series Study. Ear Nose Throat J..

[23] Nutting C., Finneran L., Roe J., Sydenham M.A., Beasley M., Bhide S., Boon C., Cook A., De Winton E., Emson M. (2023). Dysphagia-optimised intensity-modulated radiotherapy versus standard intensity-modulated radiotherapy in patients with head and neck cancer (DARS): A phase 3, multicentre, randomised, controlled trial. Lancet Oncol..

[24] Cardoso R.C., Kamal M., Zaveri J., Chambers M.S., Gunn G.B., Fuller C.D., Lai S.Y., Mott F.E., McMillan H., Hutcheson K.A. (2021). Self-Reported Trismus: Prevalence, severity and impact on quality of life in oropharyngeal cancer survivorship: A cross-sectional survey report from a comprehensive cancer center. Support. Care Cancer.

[25] van Hinte G., Sancak T., Weijs W.L.J., Merkx M.A.W., Leijendekkers R.A., Nijhuis-van der Sanden M.W.G., Takes R., Speksnijder C.M. (2021). Effect of elective neck dissection versus sentinel lymph node biopsy on shoulder morbidity and health-related quality of life in patients with oral cavity cancer: A longitudinal comparative cohort study. Oral Oncol..

[26] Jimenez J.E., Nilsen M.L., Gooding W.E., Anderson J.L., Khan N.I., Mady L.J., Wasserman-Wincko T., Duvvuri U., Kim S., Ferris R.L. (2021). factors associated with patient-reported quality of life outcomes after free flap reconstruction of the oral cavity. Oral Oncol..

[27] Bozec A., Majoufre C., De Boutray M., Gal J., Chamorey E., Roussel L.M., Philouze P., Testelin S., Coninckx M., Bach C. (2020). Oral and oropharyngeal cancer surgery with free-flap reconstruction in the elderly: Factors associated with long-term quality of life, patient needs and concerns. A GETTEC cross-sectional study. Surg. Oncol..

[28] Larson A.R., Han M., Webb K.L., Ochoa E., Stanford-Moore G., El-Sayed I.H., George J.R., Ha P.K., Heaton C.M., Ryan W.R. (2021). Patient-Reported Outcomes of Split-Thickness Skin Grafts for Floor of Mouth Cancer Reconstruction. ORL J. Otorhinolaryngol. Relat. Spec..

[29] Monte L.E.F.D., Simão I.C., Reis Junior J.R.D., Leal P.D.C., Dibai Filho A.V., Oliveira C.M.B., Moura E.C.R. (2024). Evolution of the quality of life of total laryngectomy patients using electrolarynx. Rev. Assoc. Med. Bras..

[30] Souza F.G.R., Santos I.C., Bergmann A., Thuler L.C.S., Freitas A.S., Freitas E.Q., Dias F.L. (2020). Quality of life after total laryngectomy: Impact of different vocal rehabilitation methods in a middle income country. Health Qual. Life Outcomes.

[31] Andreassen R., Jönsson B., Hadler-Olsen E. (2022). Oral health related quality of life in long-term survivors of head and neck cancer compared to a general population from the seventh Tromsø study. BMC Oral Health.

[32] Scott S.I., Kathrine Ø Madsen A., Rubek N., Charabi B.W., Wessel I., Fredslund Hadjú S., Jensen C.V., Stephen S., Patterson J.M., Friborg J. (2021). Long-term quality of life & functional outcomes after treatment of oropharyngeal cancer. Cancer Med..

[33] Deng J., Murphy B.A., Niermann K.J., Sinard R.J., Cmelak A.J., Rohde S.L., Ridner S.H., Dietrich M.S. (2022). Validity Testing of the Head and Neck Lymphedema and Fibrosis Symptom Inventory. Lymphat. Res. Biol..

[34] Harrowfield J., Isenring E., Kiss N., Laing E., Lipson-Smith R., Britton B. (2021). The Impact of Human Papillomavirus (HPV) Associated Oropharyngeal Squamous Cell Carcinoma (OPSCC) on Nutritional Outcomes. Nutrients.

[35] Ramalingam K., Yadalam P.K., Ramani P., Krishna M., Hafedh S., Badnjević A., Cervino G., Minervini G. (2024). Light gradient boosting-based prediction of quality of life among oral cancer-treated patients. BMC Oral Health.

[36] Balaji H., Aithal V.U., Varghese J.J., Devaraja K., Kumar A.N.N. (2024). Agreement between patient-reported and clinician-rated speech and swallowing outcomes—Understanding the trend in post-operative oral cavity cancer patients. Oral Oncol..

[37] Tuomi L., Karlsson T. (2021). Voice Quality, Function, and Quality of Life for Laryngeal Cancer: A Prospective Longitudinal Study Up to 24 Months Following Radiotherapy. Ear Nose Throat J..

[38] Rogers S.N., Waylen A.E., Thomas S., Penfold C., Pring M., Waterboer T., Pawlita M., Hurley K., Ness A.R. (2020). Quality of life, cognitive, physical and emotional function at diagnosis predicts head and neck cancer survival: Analysis of cases from the Head and Neck 5000 study. Eur. Arch. Otorhinolaryngol..

[39] Zivkovic A., Jotic A., Dozic I., Randjelovic S., Cirkovic I., Medic B., Milovanovic J., Trivić A., Korugic A., Vukasinović I. (2024). Role of Oxidative Stress and Inflammation in Postoperative Complications and Quality of Life After Laryngeal Cancer Surgery. Cells.

[40] Grant S.R., Hutcheson K.A., Ye R., Garden A.S., Morrison W.H., Rosenthal D.I., Brandon Gunn G., Fuller C.D., Phan J., Reddy J.P. (2020). Prospective longitudinal patient-reported outcomes of swallowing following intensity modulated proton therapy for oropharyngeal cancer. Radiother. Oncol..

[41] Jia L., Yan C., Liu R., He P., Liu A., Yang F., Huangfu H., Zhang S. (2025). Early application value of flexible laryngoscope swallowing function assessment in patients after partial laryngectomy. Sci. Rep..

[42] Aggarwal P., Hutcheson K.A., Garden A.S., Mott F.E., Lu C., Goepfert R.P., Fuller C.D., Lai S.Y., Gunn G.B., Chambers M.S. (2021). Determinants of patient-reported xerostomia among long-term oropharyngeal cancer survivors. Cancer.

[43] Hajdú S.F., Wessel I., Dalton S.O., Eskildsen S.J., Johansen C. (2022). Swallowing Exercise During Head and Neck Cancer Treatment: Results of a Randomized Trial. Dysphagia.

[44] Zhu X., Liu M., Zong M., Chen Q., Wang J. (2022). Effect of three tongue needles acupoints Lianquan (CV23) and Hegu (LI4) combined with swallowing training on the quality of life of laryngeal cancer patients with dysphagia after surgery. J. Tradit. Chin. Med..

[45] Karlsson T., Tuomi L., Finizia C. (2022). Effect of voice rehabilitation following radiotherapy for laryngeal cancer—A 3-year follow-up of a randomised controlled trial. Acta Oncol..

[46] Jansen F., Eerenstein S.E.J., Cnossen I.C., Lissenberg-Witte B.I., de Bree R., Doornaert P., Halmos G.B., Hardillo J.A.U., van Hinte G., Honings J. (2020). Effectiveness of a guided self-help exercise program tailored to patients treated with total laryngectomy: Results of a multi-center randomized controlled trial. Oral Oncol..

[47] Johansson M., Finizia C., Persson J., Tuomi L. (2020). Cost-effectiveness analysis of voice rehabilitation for patients with laryngeal cancer: A randomized controlled study. Support. Care Cancer.

[48] Li W.X., Dong Y.B., Lu C., Bradley P.J., Liu L.F. (2022). Survival and swallowing function outcome impact factors analysis of surgery-oriented comprehensive treatment for hypopharyngeal cancer in a series of 122 patients. Ear Nose Throat J..

[49] Theurer J.A., Martino R., Jovanovic N., de Almeida J.R., Goldstein D.P., Fung K., Yoo J., MacNeil S.D., Winquist E., Hammond J.A. (2025). Impact of Transoral Robotic Surgery Versus Radiation on Swallowing Function in Oropharyngeal Cancer Patients: A Sub-Study From a Randomized Trial. Head Neck.

[50] Nakai M.Y., Menezes M.B., de Carvalho J.V.B.G., Dias L.P.M., de Barros Silva L.A., Tenório L.R., Gonçalves A.J. (2021). Quality of life after Supracricoid Partial Laryngectomy. J. Otolaryngol. Head Neck Surg..

[51] Sterne J.A.C., Savović J., Page M.J., Elbers R.G., Blencowe N.S., Boutron I., Cates C.J., Cheng H.Y., Corbett M.S., Eldridge S.M. (2019). RoB 2: A revised tool for assessing risk of bias in randomised trials. BMJ.

[52] Saini R.S., Vyas R., Mosaddad S.A., Heboyan A. (2024). Efficacy of Oral Rehabilitation Techniques in Patients With Oral Cancer: A Systematic Review and Meta-Analysis. J. Surg. Oncol..

[53] Parmar A., Macluskey M., Mc Goldrick N., Conway D.I., Glenny A.M., Clarkson J.E., Worthington H.V., Chan K.K. (2021). Interventions for the treatment of oral cavity and oropharyngeal cancer: Chemotherapy. Cochrane Database Syst. Rev..

[54] Genden E.M., Kotz T., Tong C.C., Smith C., Sikora A.G., Teng M.S., Packer S.H., Lawson W.L., Kao J. (2011). Transoral robotic resection and reconstruction for head and neck cancer. Laryngoscope.

[55] Hutcheson K.A., Holsinger F.C., Kupferman M.E., Lewin J.S. (2015). Functional outcomes after TORS for oropharyngeal cancer: A systematic review. Eur. Arch. Otorhinolaryngol..

[56] Hutcheson K.A., Warneke C.L., Yao C.M.K.L., Zaveri J., Elgohari B.E., Goepfert R., Hessel A.C., Kupferman M.E., Lai S.Y., Fuller C.D. (2019). Dysphagia After Primary Transoral Robotic Surgery With Neck Dissection vs Nonsurgical Therapy in Patients With Lowto Intermediate-Risk Oropharyngeal Cancer. JAMA Otolaryngol. Head Neck Surg..

[57] Patel A.B., Hinni M.L., Pollei T.R., Hayden R.E., Moore E.J. (2015). Severe prolonged dysphagia following transoral resection of bilateral synchronous tonsillar carcinoma. Eur. Arch. Otorhinolaryngol..

[58] Tsai C.J., Jackson A., Setton J., Riaz N., McBride S., Leeman J., Kowalski A., Happersett L., Lee N.Y. (2017). Modeling Dose Response for Late Dysphagia in Patients With Head and Neck Cancer in the Modern Era of Definitive Chemoradiation. JCO Clin. Cancer Inf..

[59] Bourmand R., Olsson S.E., Fijany A. (2024). Tracheoesophageal puncture and quality of life after total laryngectomy: A systematic review and meta-analysis. Laryngoscope Investig. Otolaryngol..

[60] Boyle K., Jones S. (2022). Functional outcomes of early laryngeal cancer—Endoscopic laser surgery versus external beam radiotherapy: A systematic review. J. Laryngol. Otol..

[61] Banda K.J., Chu H., Kao C.C., Voss J., Chiu H.L., Chang P.C., Chen R., Chou K.R. (2021). Swallowing exercises for head and neck cancer patients: A systematic review and meta-analysis of randomized control trials. Int. J. Nurs. Stud..

[62] McDonald C., Kent S., Schache A., Rogers S., Shaw R. (2023). Health-related quality of life, functional outcomes, and complications after sentinel lymph node biopsy and elective neck dissection in early oral cancer: A systematic review. Head Neck.

[63] Wulff N.B., Højager A., Wessel I., Dalton S.O., Homøe P. (2021). Health-Related Quality of Life Following Total Laryngectomy: A Systematic Review. Laryngoscope.

[64] Zahavi D., Weiner L. (2020). Monoclonal Antibodies in Cancer Therapy. Antibodies.

